# Endoplasmic reticulum stress increases exosome biogenesis and packaging relevant to sperm maturation in response to oxidative stress in obese mice

**DOI:** 10.1186/s12958-022-01031-z

**Published:** 2022-11-21

**Authors:** Yangyang Li, Wenzhen Zhao, Rong Fu, Zhuoyao Ma, Yanqin Hu, Yue Liu, Zhide Ding

**Affiliations:** 1 Department of Histology, Embryology, Genetics and Developmental Biology, Shanghai Key Laboratory for Reproductive Medicine, Shanghai Jiao Tong University School of Medicine, 200025 Shanghai, China; 2 Department of Histology and Embryology, School of Basic Medical Science, Dali University, 671000 Dali, Yunnan, China; 3 Department of Core Facility of Basic Medical Sciences, Shanghai Jiao Tong University School of Medicine, 200025 Shanghai, China; 4 Department of Histology, Embryology, Genetics and Developmental Biology, Shanghai Jiao Tong University School of Medicine, No.280, Chongqing Road (South), 200025 Shanghai, China

**Keywords:** Oxidative stress, ER stress, Exosome, Epididymal epithelial cells (EECs), Comparative proteomics, Male fertility

## Abstract

**Background:**

Mammalian sperm maturation in the epididymis is mainly modulated by exosomes that are secreted into the epididymal lumen from epididymal epithelial cells (EECs). Exposure to oxidative stress (OS) resulting from being fed a high fat diet (HFD) reduces sperm fertility, which is one of the cause inducing male infertility. Thus, we hypothesize that stress-induced changes in exosome content play a critical role in mediating this detrimental process.

**Methods:**

An obese mouse model was established by feeding a HFD. Then oxidative stress status was measured in the mouse caput epididymis, epididymal fluid and spermatozoa. Meanwhile, epididymis-derived purified exosomes were isolated and validated. Subsequently, liquid chromatography tandem mass spectrometry (LC-MS) was used to perform proteomic analysis of purified exosomes. Gene Ontology (GO) analysis was performed along with pathway enrichment to identify differentially expressed proteins (DEPs).

**Results:**

Two hundred and two DEPs mostly related to endoplasmic reticulum (ER) function were identified in the exosomes separated from the epididymis of control mice and obese mice. The ER stress and CD63 (an exosome marker), both increased in the caput epididymis of obese mice. Furthermore, an in vitro study showed that palmitic acid (PA), an-oxidative stress inducer, increased exosome biogenesis and secretion in the EECs.

**Conclusion:**

Oxidative stress in the epididymal microenvironment induces ER stress in the EECs. This effect alters the epididymis-derived exosome content, profile and amounts of their differentially expressed ER proteins. Such changes may affect exosome biogenesis and cargo packaging, finally leading to abnormalities in sperm maturation and fertility.

**Supplementary Information:**

The online version contains supplementary material available at 10.1186/s12958-022-01031-z.

## Background

In mammals, spermatozoa released from the testis are immotile and incapable of mediating fertilization. In order to become competent, they need to undergo the epididymal mature procedure to be able to mediate fertilization [1, 2]. This critical step involves sperm maturation process which occurs in the epididymis. It is the first organ that spermatozoa encounter when they leave the testis. Sperm epididymal maturation is a necessary condition for further sperm capacitation and achieving fertilizing competence of the oocytes in the female reproductive tract. The most prominent feature of sperm maturation is no de novo gene transcription and protein synthesis in spermatozoa, so that this process definitely depends on the interaction between the sperm and the epididymal microenvironment. The epididymal microenvironment mainly consists of luminal fluid, in which spermatozoa receive epididymal epithelial cells (EECs) bearing derived molecules or signals critical for regulating sperm maturation and maintaining sperm quality [2–4]. The maintenance of the epididymal microenvironment relies on the secretory function or status of EECs [2–4]. As reported, EECs secrete a kind of membrane-bound vesicles, called epididymosomes containing multiple proteins and non-coding RNAs, which play crucial roles in the maintenance of the epididymal microenvironment and the communication with spermatozoa [2].

Exosome is one kind of an extracellular vesicle that has a diameter of 30 to 150 nm. It plays an important role in cell communication, immune responsiveness, metabolic regulation and tumor progression [5–7]. Epididymosomes are exosomes derived from the epididymis which are synthesized in EECs and secreted into the epididymal lumen by the apocrine pathway [2, 8, 9]. There are quite a few reports that document exosome involvement in mediating sperm maturation. Zhou, et al. found that the proteins in epididymosomes can be transferred to the post-acrosomal domain of the mouse sperm, indicating that epididymosome-sperm interaction may contribute to the sperm maturation and sperm-oocyte fusion [10]. The proteomic analysis of the epididymosomes isolated from the caput and cauda epididymis in mice respectively showed that both protein components and the characteristics of epididymosomes were significantly different between the two anatomic regions, suggesting that the epididymosomes stemmed from EECs in various epididymal sections may have some different functions [9]. Moreover, in the clinic, the exosomes derived from the seminal plasma of severe asthenozoospermia patients significantly decreased the progressive motility of normal sperm after co-incubated in vitro, further indicating its important role in regulating sperm function [11]. On the other hand, the contents of epididymosomes, such as proteins and non-coding RNAs, were altered or modified in response to the environmental stresses. Such changes can effectively regulate sperm maturation through sperm interaction with epididymosomes [2].

Currently, there are almost 15% couples suffering from infertility all over the world, and among these couples, approximately 50% of the cases stem from male infertility [12, 13]. Overwhelming evidence suggests that oxidative stress (OS) is a major cause of sperm dysfunction and it plays a significant role in the etiology of male infertility owing to its impairment of both the structural and functional integrity of spermatozoa [14–16]. The causes of OS associated male infertility involve a wide range of factors including genetics, epigenetics, environmental and lifestyle-related factors [13, 17]. Pathological processes such as genitourinary tract infection and varicocele are the widely accepted causes of oxidative stress, while unhealthy lifestyle-related factors such as psychological stress, obesity and advanced paternal age are also recognized as possible causes of oxidative stress, which can lead to male infertility or subfertility [17–19]. More importantly, the evidence demonstrates that the exosomal cargos and the exosome-sperm communication have been chronically changed by stress or dietary constraints in a number of animal studies [20, 21], but the mechanism of the exosome packaging and content change in the EECs induced by OS has not been reported until now.

Recent studies dealing both with obese mice on a high-fat diet and in the high-BMI males showed that obesity can increase OS and the DNA damage, and also decrease the sperm motility [22, 23]. Thus, the excessive ROS, which was induced by obesity in the epididymal microenvironment, may be one of major causes for sperm dysfunction and male infertility [18]. In the current study, we identified increases in epididymal OS and sperm ROS accompanied declines in male fertility in obese mice fed a high fat-diet. The epididymis-derived exosomes in these mice fed a high fat diet had a protein expression profile that was resolved using the aforementioned proteomic procedures. It was different than that in the normal weight control mice fed instead a control diet. Especially, we found that endoplasmic reticulum (ER) stress induced several protein changes in the exosomes and promoted exosome biogenesis in the EECs. Meanwhile, our study initially elucidated the mechanism of OS-associated ER stress in the caput epididymis, which can induce different exosome cargos and impair sperm quality during epididymal maturation.

## Materials and methods

### Animals

All the 3 weeks old wild type male mice (C57BL/6) were purchased from Shanghai Laboratory Animal Center. They were fed and housed in the Animal Center of Jiao Tong University Medical School with standard lighting and ad libitum access to drinking water. After a week of acclimatization, the mice were fed a 45% high-fat diet (Medicience, Jiangsu, China) to establish an obesity model (obese mice) while the control group was fed a normal diet (control mice). Their oxidative stress indices were measured 12 weeks after the two groups had been continuously fed the high-fat and normal diets. On the other hand, the mice used for the isolation of primary epididymal epithelial cells (EECs) were procured at 2 weeks and immediately used in the experiments.

Animal experiments were conducted according to the International Guiding Principles for Biomedical Research Involving Animals, as promulgated by the Society for the Study of Reproduction. This research program was approved by the ethics committee of Shanghai Jiao Tong University School of Medicine (No. A2015-034).

### Sperm parameter assay

The cauda epididymides of the obese mice and control mice were separated and cut into pieces in the Tyrode’s Buffer (Sigma, T2397, USA) preheated at 37 ℃. Tissue suspensions were incubated for 15 min at 37 ℃ to release the sperm. Then, the computer-assisted sperm analysis (CASA) system was used to evaluate the sperm suspension concentration and motility analyses (Hamilton Thorne, USA).

### Oxidative stress detection

The mouse caput epididymis and epididymal fluid were detached and extracted, respectively, after being fed for 12 weeks. For the GSH/GSSG measurement of caput epididymis, the tissue was homogenized in the 5% sulfosalicylic acid (SSA) and then centrifuged at 8,000 xg for 10 min at room temperature. The supernatant was evaluated by following the protocol of the GSSG/GSH Quantification Kit II (Dojindo, G263, Japan). Meanwhile, the whole mouse epididymis was separated in PBS (Phosphate Buffer Saline) and cut into pieces in a tube and then set on ice to release the epididymal fluid for 20 min. After centrifuged at 12,000 xg for 10 min at 4 ℃, the precipitate was discarded and the oxidative stress level of the epididymal fluid was measured with the Lipid Peroxidation MDA Assay Kit (Beyotime, China). The sperm suspension was obtained as described above and then labeled with DCFH-DA Dye (Dojindo, G252, Japan) and subsequently flow cytometry (CytoFlex LX, USA) was used to measure the ROS levels.

### Isolation of epididymis-derived exosomes

The supernatant of epididymal fluid was released as described above and the exosome isolation reagent was added in the proportion of 1:5 (Yeasen, 41,202-A, China), and then the mixture was incubated for 2 h at 4 ℃. After centrifuged at 10,000 xg for 1 h at 4 ℃, the supernatant was discarded and the pellet was resuspended with PBS. An aliquot of the extracted exosomes was immediately validated by transmission electron microscopy (TEM) analysis and the rest was stored at -80 ℃ for later experiments.

### Transmission electron microscopy (TEM)

The exosome suspension was added to 2% glutaraldehyde and incubated at 4 ℃ overnight. The mixture was added to copper grids for 10 min at room temperature and then the solution was removed. Next, the samples adsorbed on the copper grids were counterstained with uranyl acetate for 5 min. Finally, the samples were analyzed by transmission electron microscopy (FEI-Talos-120, Thermo Fisher, USA).

### Nanoparticle tracking analysis (NTA)

The Zetaview particle potentiometric titration and particle size analyzer were used to perform nanoparticle tracking analysis (Particle Metrix, Germany). The exosome suspension was diluted by PBS and illuminated with 405 nm emission light.

### Proteomic analysis of epididymis-derived exosomes and bioinformatics analysis of differentially expressed proteins (DEPs)

The epididymis-derived exosomes of five control mice and five obese mice were collected in the PBS, respectively, and then liquid chromatography tandem mass spectrometry (LC-MS) were performed as described [24]. Proteome Discoverer 2.2 software (Thermo Fisher, USA) was used to search spectral analyses results. The credibility of the identified Peptide Spectrum Matches (PSMs) had to be above 99% and the identified protein had to contain at least 1 unique peptide. The false discovery rate (FDR) of identified PSMs and protein was performed as no more than 0.01. Then, the DEPs were mapped into the Gene Ontology database for GO analysis.

### Isolation, culture and drug treatment of mouse primary epididymal epithelial cells

The caput epididymis of 2-week-old male mice was separated in PBS with 1% penicillin/streptomycin (Gibco, 15,140, USA), and cut into pieces. Then collagenase I (100U/ml, Gibco, 17,100, USA) was added to the tissue suspension and incubated at 37 ℃ for 1–2 h. When the tissue was digested completely, the suspension was centrifuged at 200 xg for 1 min at room temperature and the supernatant was discarded. The pellet was washed with 500 µl PBS and centrifuged at 200 xg for 1 min twice. Finally, the pellet was resuspended in the complete F12 medium with 10% FBS (Gibco, A3160801, USA) containing 1% penicillin/streptomycin and then immediately cultured in the incubator with 5% CO_2_ at 34 ℃. After deposited for 45 min, the supernatant containing EECs was collected and transferred to a new 10 cm dish and continuously cultured at the same condition as described above. When the density of EECs reached 80%, the palmitic acid (PA) at a final concentration of 200 µM was added to the experimental group in the culture medium, while only culture medium was added to the control group. Western blot analysis was performed after the cells were treated for 48 h.

### Isolation of EECs-derived exosomes

For the collection of the EECs-derived exosomes, the cells were cultured with the complete F12 medium containing 10% FBS (SBI, EXO-FBS-50 A-1, USA) and 1% penicillin/streptomycin. The supernatant of primary EECs treated with PA or culture medium (control) was collected and centrifuged at 3000 xg for 15 min at room temperature. Then the supernatant was diluted with the exosome isolation reagent in a ratio of 1:4 (Yeasen, 41201ES50, China) and subsequently the extraction process was the same as that described for the isolation of epididymis-derived exosomes.

### Protein sample preparation and Western blot

For the detection of exosome markers, the exosome suspension was added to an equal volume of RIPA and incubated at 4 ℃ for 30 min. For the preparation of tissue protein samples, the caput epididymis of the obese mice and control mice were collected in the RIPA with protease inhibitors and cut into pieces and then incubated at 4 ℃ for 30 min. For the cell protein sample preparation, the cells treated with or without PA for 48 h were washed with PBS twice and harvested in the RIPA with protease inhibitors and incubated at 4 ℃ for 30 min. All the protein samples were added to 5x SDS loading buffer, and boiled at 100 ℃ for 10 min and centrifuged at 12,000 xg for 10 min. The supernatants were separated on a 10% SDS-PAGE gel and transferred to the PVDF membrane (Mlipore, USA) by the Bio-Red Trans-Blot (Bio-Red, USA). Then the membrane was blocked by the Protein Free Rapid Blocking Buffer (Epizyme, China) for 30 min at room temperature. The primary antibodies were added and incubated overnight at 4℃, including exosome markers TSG101 (1:2000, abcam, ab125011, UK), CD9 (1:2000, abcam, ab92726, UK), CD63 (1:2000, abcam, ab229912, UK), Flotillin 1 (1:2000, abcam, ab133497, UK), and the differential expression protein in proteomic analysis such as PDIA3 (1:2000, CST, 2881 S, USA), RRBP1 (1:2000, abcam, ab95983, UK) and CRELD2 (1:2000, Santa Cruze, sc-365,168, USA), and the ER stress markers PERK (1:1000, abcam, ab229912, UK) and IRE1α (1:1000, CST, 32,947, USA). After washing with TSBT, the membranes were incubated with the secondary antibodies goat anti-rabbit or goat anti-mouse at room temperature for 1 h. Then the membranes were washed with TBST again and detected by the Light Chemiluminescence Kit (Epizyme, China). Finally, the protein band intensity was quantified with ImageJ (USA) software.

### Immunohistochemistry (IHC)

The caput epididymis was fixed in Bouin’s solution overnight, and dehydrated in a gradient ethanol series. After hyalinized by xylene, the tissue was embedded with paraffin and sectioned. The paraffin-embedded sections were dewaxed by xylene and hydrated by gradient ethanol. Then the sections were boiled for 15 min in the 10 mM citrate buffer at pH 6.0 for antigen retrieval. After incubated for 10 min in the 3% H_2_O_2_ to block endogenous peroxidase, the sections were further blocked by 5% BSA. Subsequently, the sections were incubated with the primary antibody overnight at 4 ℃ as follows: RRBP1, CRELD2 and PDIA3 (1:200 dilution). Meanwhile, normal rabbit IgG or normal mouse IgG was employed as controls. After that, the section was incubated with the second antibody either against rabbit or against mouse for 1 h at room temperature. Then the sections were stained with DAB and hematoxylin, respectively and finally the pictures were captured under the microscope (OLYMPUS, BX53, Japan).

### Immunofluorescence (IF)

The primary EECs seeded on the slides were fixed with 4% paraformaldehyde (PFA) for 30 min and permeabilized with 0.5% Triton-100 for 15 min. Then the cells were blocked by 5% BSA dissolved in the PBS for 1 h at room temperature. The cells were co-incubated with the primary antibodies including CK-18 (1:500, the marker of EECs, Abcam, USA) and Vimentin (1:200, the marker of mechanocyte, R&D systems, USA) overnight at 4 ℃. After washed by PBS, the cells were incubated with the secondary antibodies Donkey anti-rabbit (Biotium, USA) and Donkey anti-mouse (Biotium, USA) for 1 h at room temperature in the dark. After washed with PBS, the fluorescence microscope (OLYMPUS, BX53, Japan) imaged the DAPI Fluoromount-G (Yeasen, 36308ES20, China) mounted slides.

### Statistical analysis

At least three independent repetitions were performed of all the data. The results were expressed as means ± SD. The data comparisons were examined using two-sample Student’s *t* test by software GraphPad Prism (USA), and *P* values of < 0.05 were considered significant.

## Results

### Association between obesity and oxidative stress in the epididymal microenvironment

**Fig. 1:**
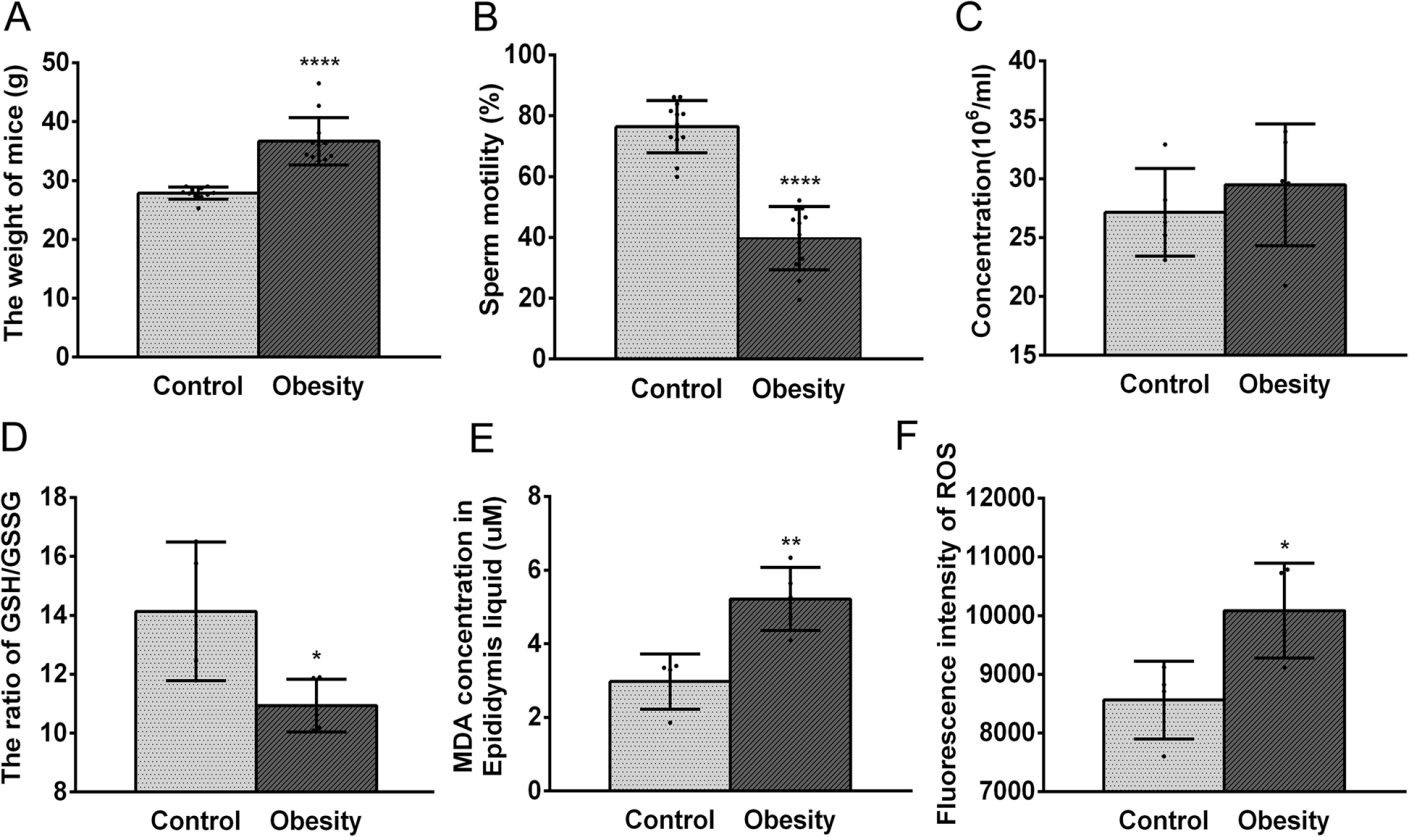
Obesity induced the oxidative stress and poor sperm quality in mouse epididymis. **A** The body weight of obese mice significantly increased compared to that in control mice. **B-C** The comparisons between normal mice and obese mice in sperm motility and concentration. **D** In the caput epididymis of the obese mice, the ratio of the GSH/GSSG decreased. **E** The concentration of MDA increased significantly in the epididymis fluid of the obese mice. **F** The level of ROS increased in the sperm of obese mice. All data were presented as mean ± SD, *P < 0.05, **P < 0.01, ****P < 0.0001

Starting at 4 weeks of age, male mice were fed either a high-fat diet (obese mice) or normal diet (control mice). After being fed for 12 weeks, the body weight of obese mice significantly increased compared with that of the control mice (Fig. 1 A). For the sperm parameters, the sperm motility in obese mice significantly decreased compared with that in control mice, but no significant change in sperm concentration was observed (Fig. 1B, C). These features in obese mice were in agreement with our previous reports [25].

The oxidative stress in the epididymal microenvironment was evaluated based on measurements of the ratio of GSH to GSSG in the caput epididymis and the MDA (malondialdehyde) concentration in the epididymal fluid. The results showed that the ratio of GSH to GSSG was significantly declined (Fig. 1D) while the MDA concentration increased (Fig. 1E) in obese mice, which indicated that obesity can induce OS in the epididymis microenvironment. Accordingly, the sperm of obese mice exhibited a significantly higher ROS level than that in the control mice (Fig. 1 F). These results clearly showed that obesity induces oxidative stress in the epididymal microenvironment. This condition imposes oxidative damage that includes poor sperm quality in mice.

### Isolation and validation of exosome identity

**Fig. 2:**
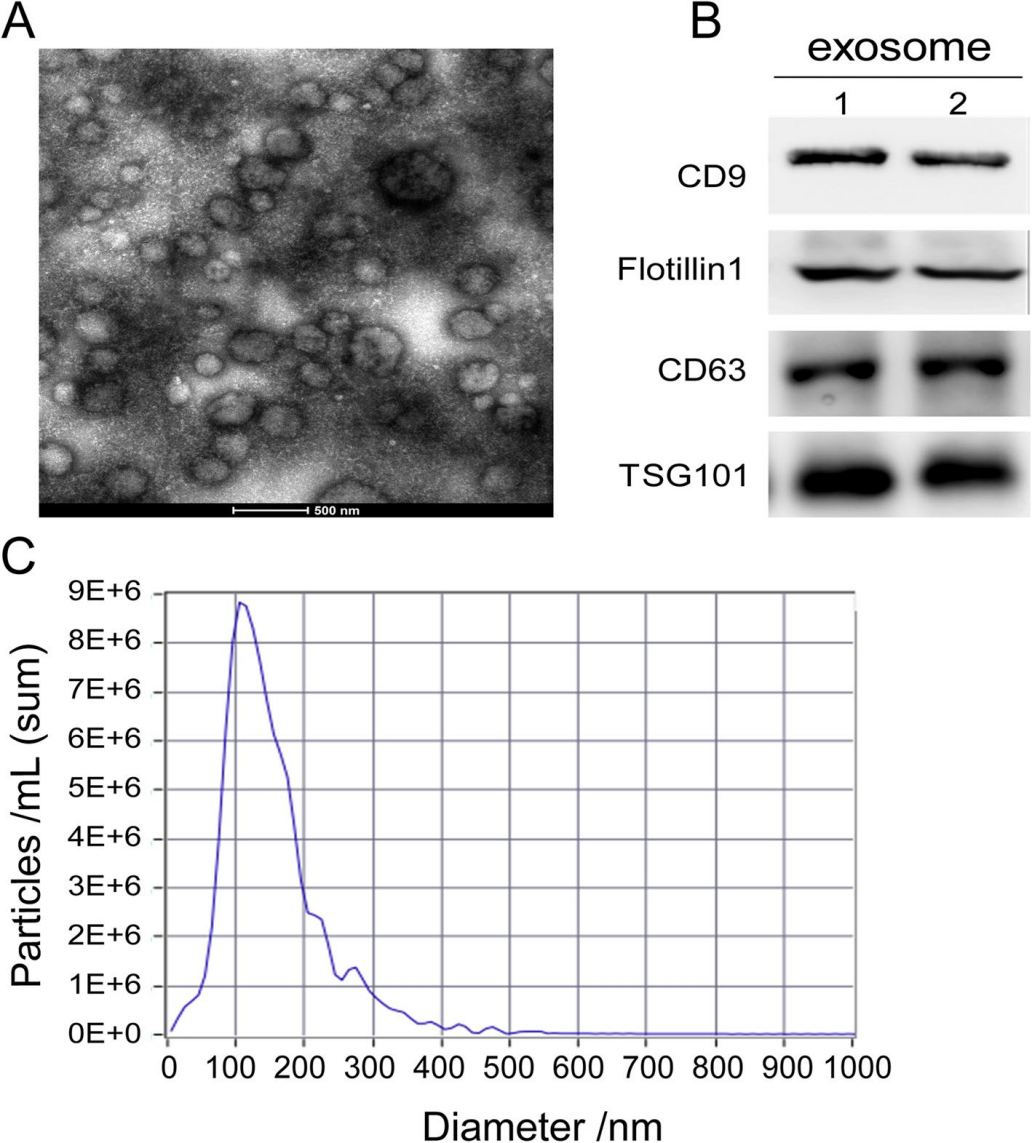
The characteristics of the purified exosomes. **A** The exosomes observed by transmission electron microscopy (TEM), Bar = 500 nm. **B** The size of the exosomes detected by NTA. **C **Identification of the CD9, Flotillin 1, CD63 and TSG101 in the exosome protein by Western blot analyses. The samples loaded in the lane1 and lane2 were exosome protein extracted from the epididymis of normal mice

Polymer-based precipitation was performed to isolate the exosomes. TEM analysis showed that the exosomes were apparent and filled with epididymal fluid (Fig. 2 A). In the experiment of NTA, almost all of exosome (96.8%) diameters were 116.3 nm and their concentration was 1.1 × 10^10^ particles per mL (Fig. 2B). This size of the isolated vesicles coincided with that defined for exosomes. The exosome molecular markers include CD9, Flotillin 1, CD63 and TSG101, and Western blot analysis detected them as being enriched in the isolated exosomes (Fig. 2 C). Therefore, our results indicated that these exosomes isolated from mouse epididymal fluid had high homogeneity and purity.

### Differential protein expression profiles of epididymal-derived exosomes in obese mice analyzed by comprehensive proteomics

**Fig. 3:**
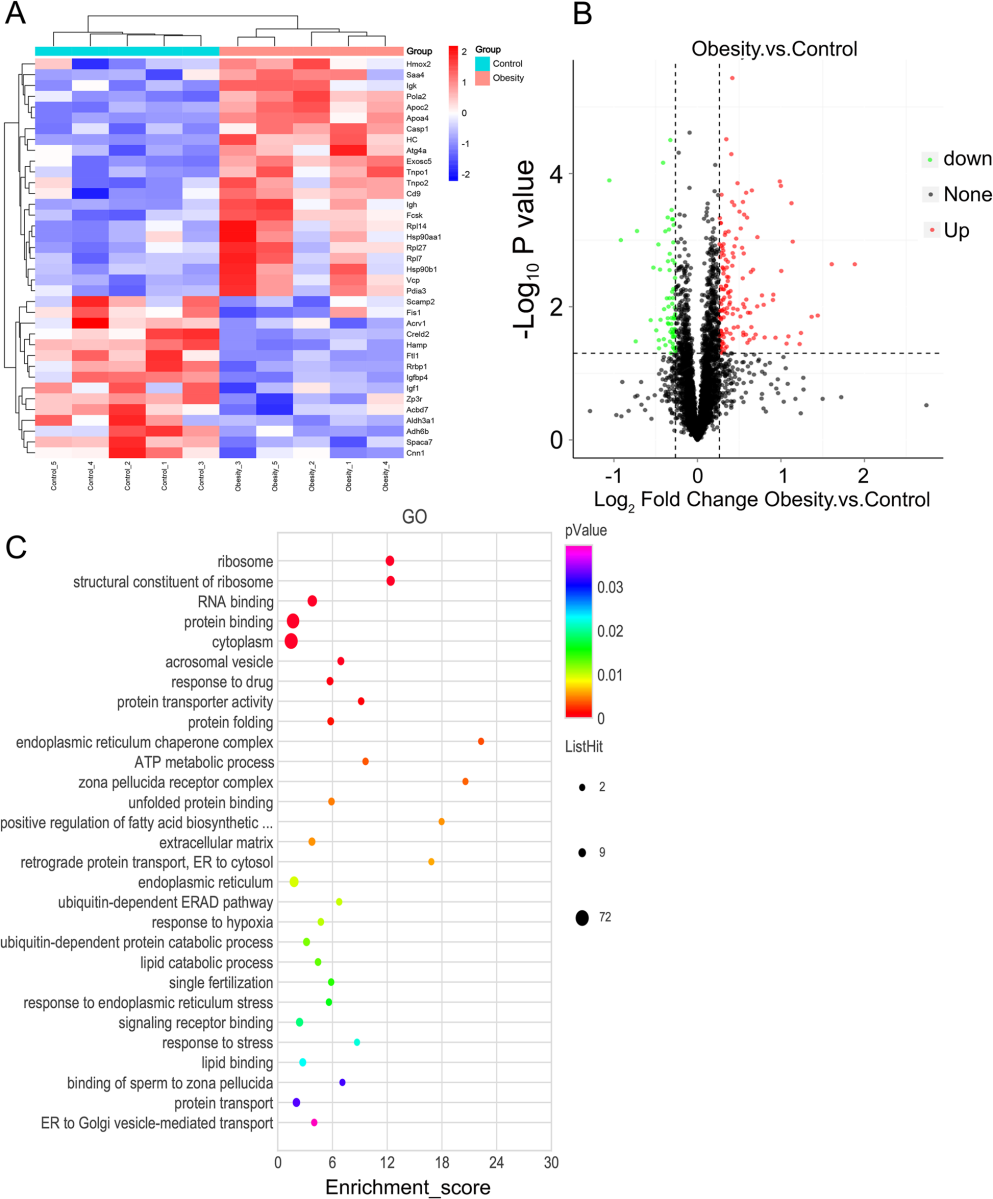
Differential protein expression profiles of epididymis-derived exosome between the control mice and obese mice. **A** The heatmap showed parts of differential expressed proteins in exosomes derived from the epididymis of obese mice and control mice. **B** Volcano plots revealed the differential proteins with 1.2-fold changes and *P* value < 0.05. Red dots represented the up-regulated proteins and green dots represented the down-regulated proteins. **C** GO analysis of the differential proteins in exosomes derived from the epididymis of obese mice and control mice

To determine protein composition and quantitation of the epididymal-derived exosomes isolated from obese mice and control mice, TMT-based proteomic analysis coupled with LC-MS/MS was performed using five individual exosome samples in each group. The analysis used 391,679 spectra and among them, there were 33,579 unique peptides. In total, 4338 proteins were identified in both groups, and 202 differentially expressed proteins (DEPs) were identified with 1.2-fold changes in protein abundances and *P* value < 0.05, including 138 proteins which were up-regulated and 64 proteins were down-regulated, respectively (Fig. 3 A, B).

The gene ontology (GO) analysis showed that the DEPs were mainly enriched in the cytoplasm function, protein transport, stress reaction, and endoplasmic reticulum (ER) function (Fig. 3 C). Meanwhile, most of these DEPs were related to the processes of immunoregulation, respond to stress, lipid metabolism, endoplasmic reticulum function and fertility (Table 1). Furthermore, the proteins enriched in ER function according to GO analysis were obviously related to “protein folding”, “ER stress” and “ER transport”. Notably, ER stress can be induced by OS and further impair ER function, such as protein secretory and vesicle transport [26–29]. Accordingly, the results of bioinformatics analyses strongly indicated that these DEPs account for the variable composition of exosomes associated with ER stress.

**Table 1:** Partial classification of the differentially expressed proteins

**Classify**	**Gene**	**Physiological processes**
Immunoregulation	Rbm14, Il1rap	innate immune response
	Casp1	regulation of inflammatory response
	Enpp1	immune response
Respond to stress	Casp1, Srp72, Ube2b, Jund,	respond to drug
	Serpina7, Aldh3a	
	Hmox2, Hsp90b1, Casp1, Aldh3a1	respond to hypoxia
	Eif2s1, Pdcd6	respond to heat
	Hmox2, Atox1	respond to oxidative stress
Lipid metabolism	Apoc2, Apoa4, Sult1e1, Acbd7, Nr5a1, Vcp	lipid binding
	Lipf, Pld3, Ces1b, Plbd1	lipid catabolic process
	Arfip2, Nr5a1, Apoa4	phospholipid binding
	Apoa4	positive regulation of lipid biosynthetic process; lipid homeostasis; lipid transport
	Lipf, Hacl1, Echdc2, Pld3, Plbd1, Ptgds	lipid metabolic process
	Vcp	lipid droplet
Endoplasmic reticulum function	Vcp, Rrbp1, Pdia3, Hsp90b1, Hsp90aa1, Eif2s1, Dnajb11	Protein processing in endoplasmic reticulum
Fertility	Ube2b	sperm axoneme assembly
	Cd9	fusion of sperm to egg plasma membrane involved in single fertilization
	Insl6, Acrbp	spermatid development
	Acrbp	sperm capacitation
	Hsp90aa1	sperm flagellum
	Insl6	flagellated sperm motility
	Insl6, Ube2b	spermatogenesis
	Fgf2, Camsap3	embryo development ending in birth or egg hatching
	Zp3r, Cct6a	binding of sperm to zona pellucida

### Expression level analyses of relevant DEPs including RRBP1, CRELD2 and PDIA3 in epididymis-derived exosomes

**Fig. 4:**
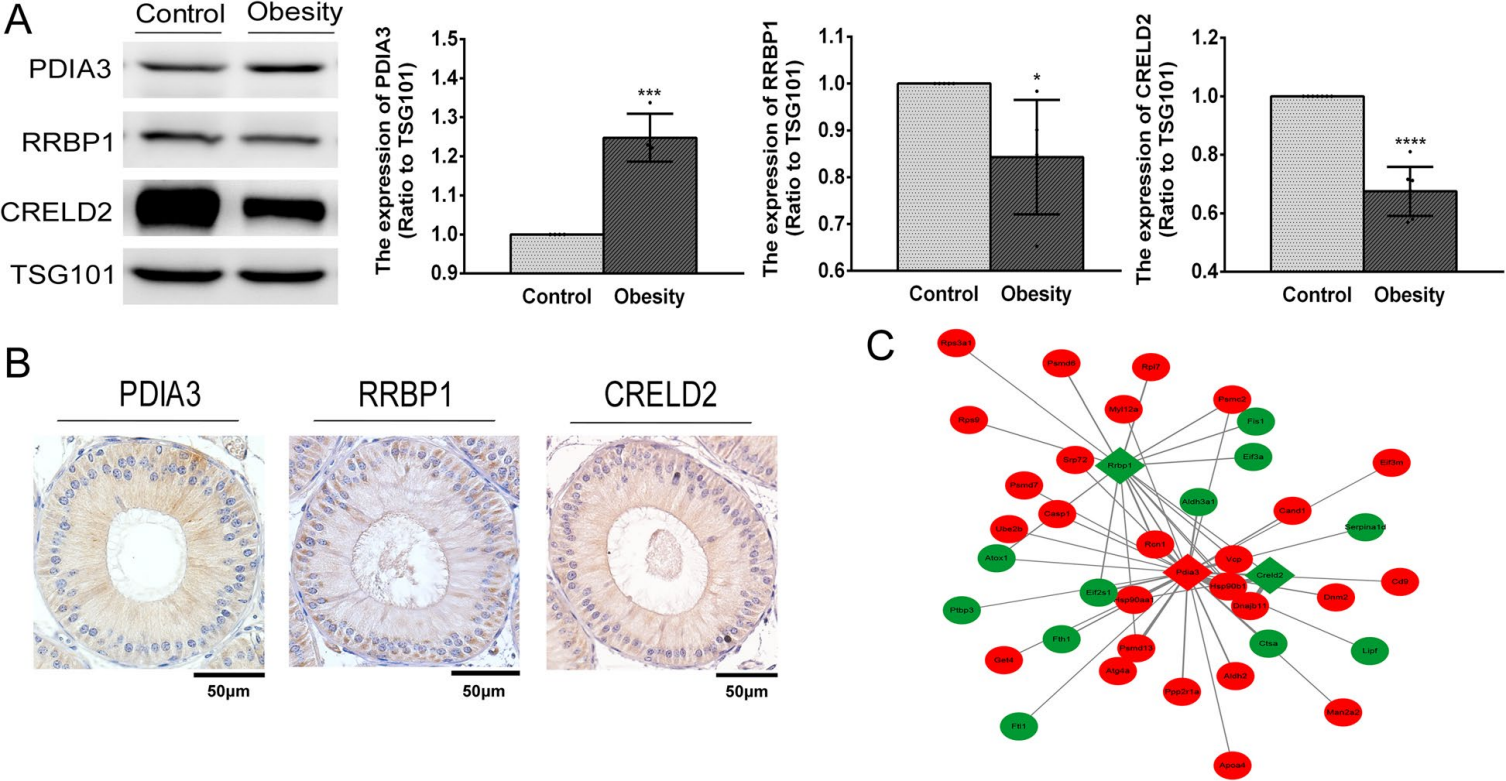
Expression levels of the candidate proteins in the epididymis-derived exosomes and caput epididymis. **A** The expression of candidate proteins PDIA3, RRBP1 and CRELD2 in the exosomes detected by Western blot analysis, and their expression levels were normalized to TSG101 expression (exosome marker). All data were shown as mean ± SD, *P < 0.05, ***P < 0.001, ****P < 0.0001. **B** The candidate proteins PDIA3, RRBP1 and CRELD2 were widely expressed in the epithelial cells lining the lumen of caput epididymis (bar = 50 μm). **C** PPI showed the relationship between the candidate proteins with the other differentially expressed proteins resolved in the proteomic data. The diamonds represent the candidate proteins and the circles represent the associated proteins. The red coloration indicates the up-regulated proteins whereas the green coloration indicates the down-regulated proteins

Several studies demonstrated that some ER proteins including RRBP1, CRELD2 and PDIA3 are related to the ER stress and involved in multiple pathological processes (Table 2). Thus, Western blot analysis was performed to evaluate the levels of these candidate proteins. In comparison to those in control mice, the levels of RRBP1 and CRELD2 in exosomes were downregulated in obese mice, whereas exosomal PDIA3 expression was upregulated, which agreed with the proteomic data (Fig. 4A). Additionally, IHC analysis showed that these candidate proteins were mainly localized in the cytoplasm of the EECs lining the lumen of the caput epididymis which mediates the protein secretion (Fig. 4B). Notably, the PPI network of the DEPs showed that these candidate proteins were closely correlated with the other DEPs involved in the regulation of the ER function (Fig. 4C). Therefore, the results of proteomic analyses indicate that the ER proteins such as RRBP1, CRELD2 and PDIA3 in the exosomes were differentially regulated by the obesity-associated OS condition in the caput epididymis and may indeed affect sperm maturation.

**Table 2:** ER stress-associated candidate genes

Protein name	Gene	Location and function	Physiopathological process
Ribosome binding protein 1	*Rrbp1*	mainly located in the ER [30, 31], critical for ribosome binding and transportation and secretion [30, 32].	involved in the tumorigenesis, including, esophageal carcinoma, bladder cancer, prostate cancer, lung cancer [29, 30, 32, 33].
Cysteine-rich with EGF-like domains 2	*Creld2*	located in the ER, identified as a novel ERS inducible gene [34].	involved in the ER-stress-mediated kidney disease and aortic aneurysm in Marfan syndrome, osteogenic differentiation [35–38].
Protein disulphide isomerase-3	*Pdia3*	mainly located in the ER [39],belongs to the protein disulphide isomerase family and catalyses the reduction, oxidation and isomerization of the substrate disulfide bond [39, 40].	involved in the assembly of MHC, the blood platelet aggregation and thrombosis, the sperm-oocyte fusion [39–42].

### Association between elevated ER stress levels and epididymis-derived exosome secretion in the caput epididymis of obese mice

**Fig. 5:**
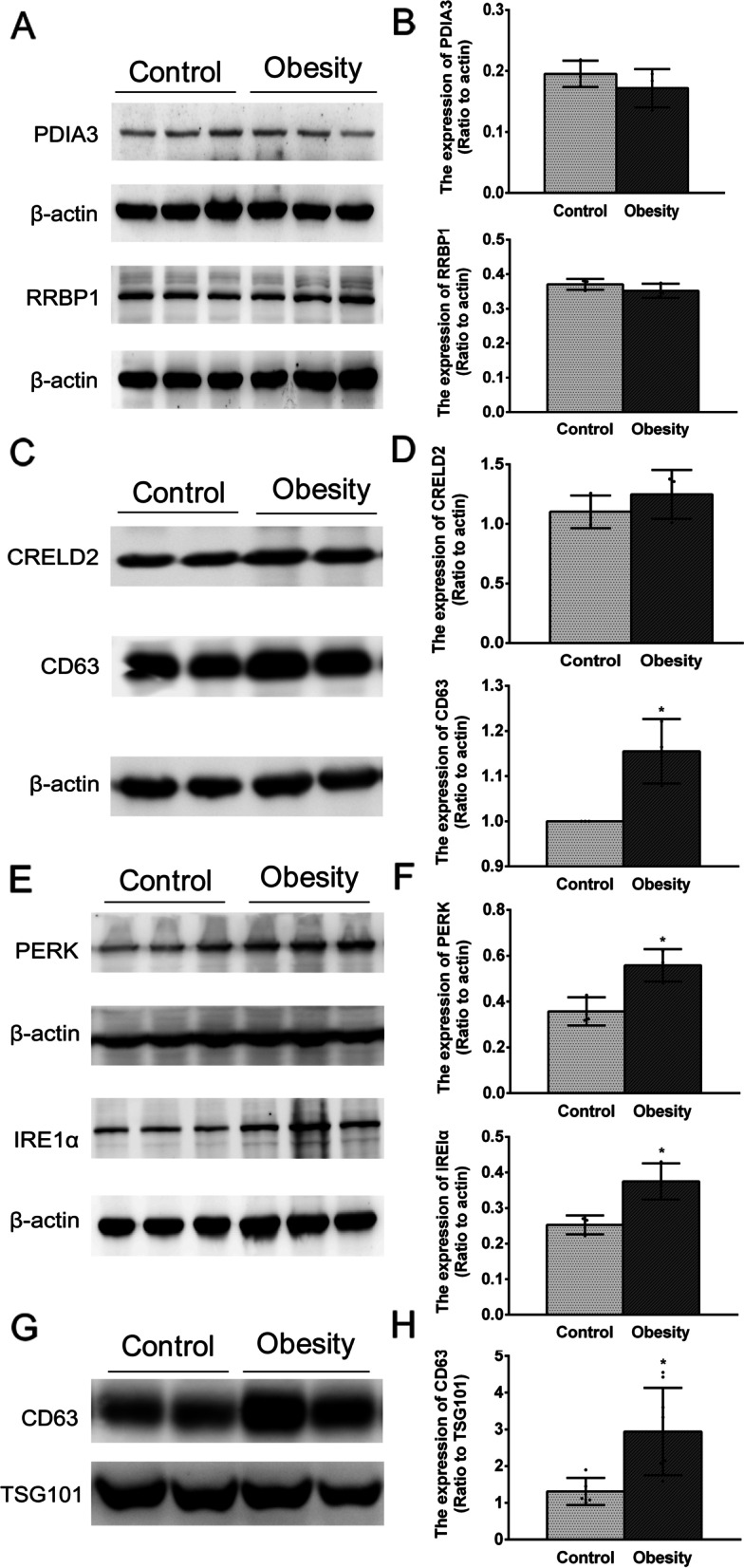
Association between increases in ER stress and exosomal expression levels in the caput epididymis of obese mice. **A-B** Representative Western blot analyses of candidate proteins PDIA3, RRBP1 in the caput epididymis of control mice and obese mice, respectively. **C-D** Representative Western blot analysis of candidate protein CRELD2 and exosome marker CD63 in the caput epididymis of control mice and obese mice, respectively. **E-F** The caput epididymis of the obese mice showed high level of ER stress with the up-regulated expression of IRE1α and PERK. **G-H** Representative Western blot analysis of exosome marker CD63 in the exosomes of control mice and obese mice, respectively. The relative quantification of the tissue proteins was normalized to β-actin and the exosome proteins were normalized to TSG101. All data were presented as mean ± SD, *P < 0.05

Although the expression patterns of RRBP1, CRELD2 and PDIA3 in the exosomes changed in obese mice, Western blot analysis showed that there were no differences in their expression levels in the caput epididymis between the control and obese mice (Fig. 5 A,B,C,D). Such inconsistency between their expression levels in the epididymis and the epididymis-derived exosomes may contribute to their predominant distribution in the ER whereas only a part of these proteins may be sorted into the exosomes. Thus, we were much more interested in the cause of the differentially expressed exosome protein packaging in the epididymal epithelium cells in response to obesity. Since the differentially expressed ER proteins in the exosomes were related to ER stress (Table 2) and several other studies also reported that ER stress can regulate the quantities of exosomes [43, 44], we speculated that the protein sorting in the ER as well as exosome biogenesis may be changed in response to ER stress.

When cells are under stress, ER stress is induced, which activates unfolding protein response (UPR) in the endoplasmic reticulum. This response plays an important role in maintaining ER homeostasis [29, 45, 46]. As reported, the ER sensors including inositol-requiring enzyme 1α (IRE1α) and protein kinase R-like ER kinases (PERK) are crucial for regulating ER stress [43, 45, 46]. Thus, in our study, high ER stress in the caput epididymis of obese mice was confirmed based on showing that the IRE1α and PERK expression levels underwent significant upregulation (Fig. 5E, F).

Meanwhile, to explore whether the exosome quantities were also altered in obese mice, we monitored the CD63 expression level, which is an exosome molecular marker in the caput epididymis. As shown in Fig. 5 C, D, G and H, the CD63 expression levels in the caput epididymis and the exosomes of obese mice were up-regulated more than those in the control mice, which indicated that the caput epididymis of obese mice secreted more exosomes compared with that in the control mice. In summary, our results suggest that increased ER stress in the caput epididymis of obese mice may induce increases in exosome biogenesis and secretion, and especially alter the composition of proteins that are involved in inducing ER stresses in obesity.

### Association between increases in EEC-derived exosome amounts and oxidative-induced ER stress

**Fig. 6:**
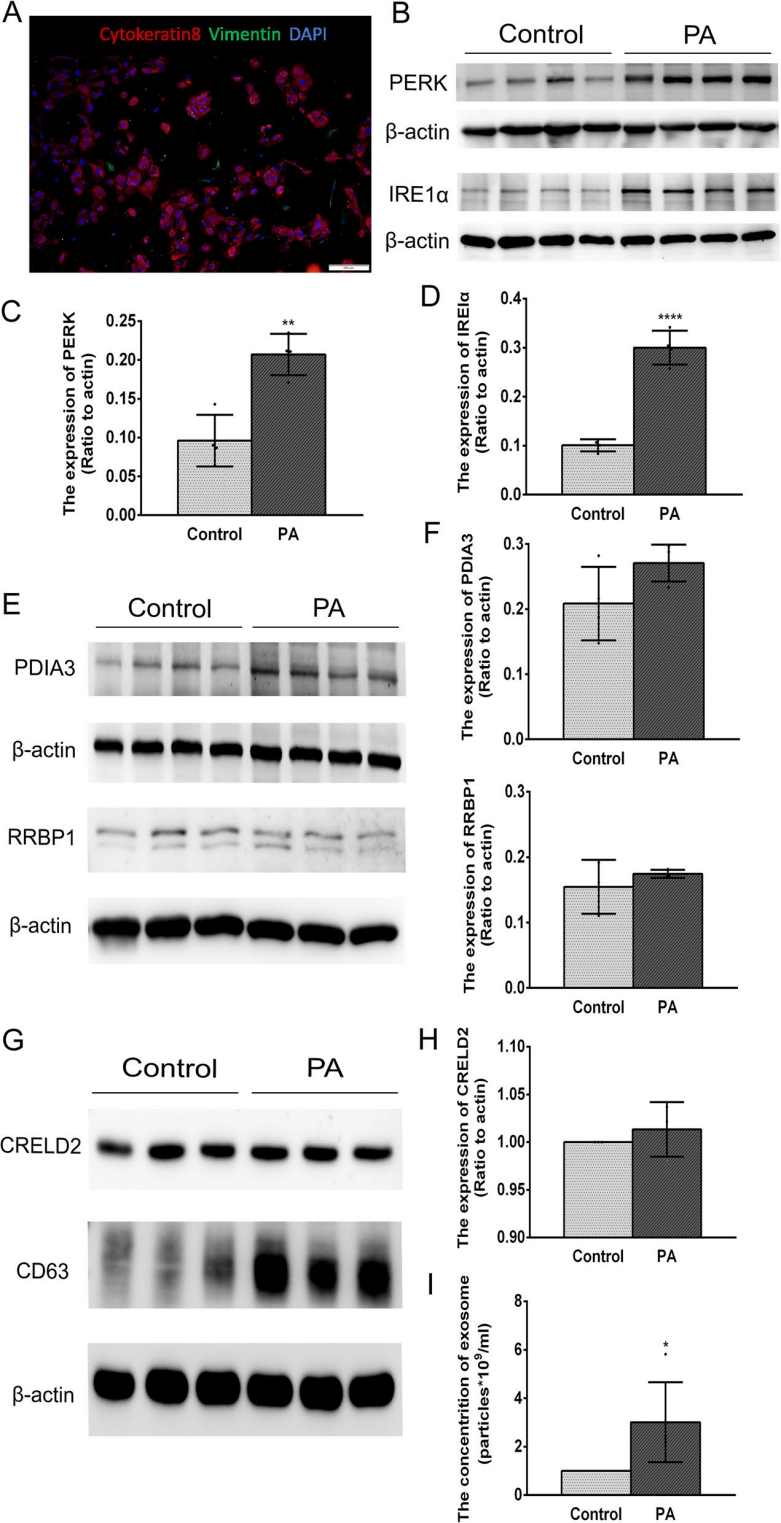
Palmitic acid (PA) significantly induces the ER stress in the primary EECs and promotes the EEC-derived exosome biogenesis in EECs. **A** Co-immunofluorescent staining of cytokeratin 8 (red) and vimentin (green) evaluated primary EECs purity. **B-D** PA significantly induced ER stress based on the upregulation of PERK and IRE1α in the EECs. **E-F** Western blot analysis detected the expression levels of the candidate proteins, PDIA3 and RRBP1, in the EECs with or without PA treatment. **G-H** Representative Western blot analyses of candidate protein CRELD2 and exosome marker CD63 in the EECs with or without PA treatment. **I** NTA determination of the concentration of the exosomes derived from the EECs. The relative quantification of the proteins was normalized to β-actin. All the data were presented as mean ± SD, *P < 0.05, **P < 0.01, ****P < 0.0001

To further probe the effect of ER stress on the exosome biogenesis in vitro, primary cultures of EECs were employed. The cell purity of primary EECs was over 85%, which was assessed by co-immunofluorescent staining of cytokeratin-8, a protein epithelial cell marker (as positive marker), and Vimentin staining, which is a protein fibroblast molecular marker (as negative control) (Fig. 6 A).

Palmitic acid (PA) is a kind of saturated fatty acid, which can induce the OS through generating high ROS levels [47–49]. In the current study, the primary EECs were treated with PA in order to induce OS. As shown in Fig. 6B, C and D, the expression levels of PERK and IRE1α (representing the level of ER stress) in EECs treated with PA significantly increased compared with those in control cells. Meanwhile, we detected the selected candidate proteins in the DEPs and CD63 expression levels in the EECs in the presence and absence of PA treatment. In agreement with the results in vivo, PA treatment did not alter the protein levels of RRBP1, CRELD2 and PDIA3 in EECs, but instead increased the expression of CD63 (Fig. 6E, F, G, H). Such CD63 upregulation in the EECs suggested an association between increases in exosome biogenesis and PA-induced ER stress. Moreover, the exosomes secreted in the primary cultures of EECs were extracted and NTA evaluated their concentration. PA significantly increased the exosome quantities (Fig. 6I). Accordingly, all these results indicated that OS-induced ER stress promotes exosome biogenesis and it affects the exosome packaging both in vivo and in vitro.

## Discussion

**Fig. 7:**
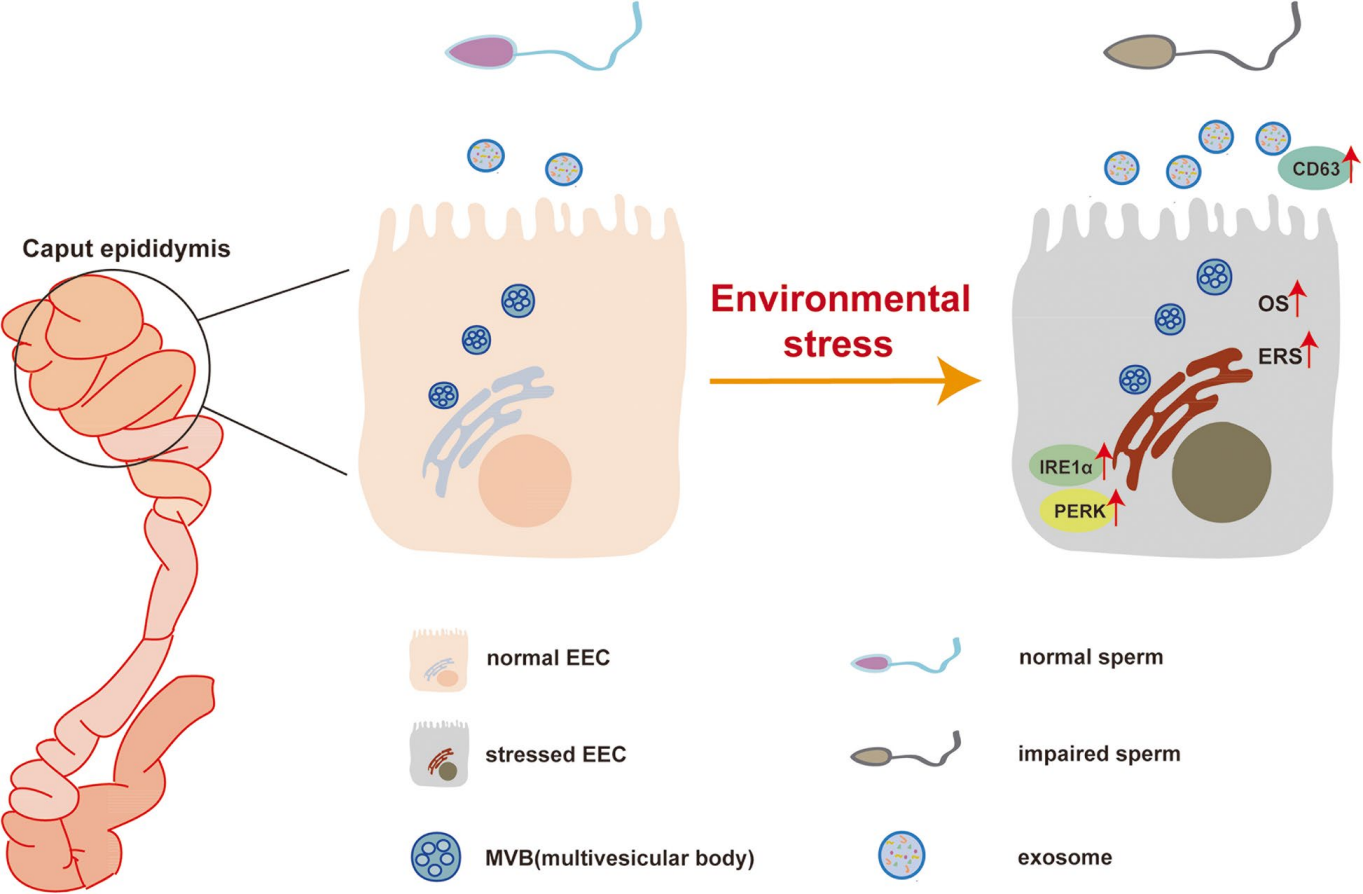
Model describing how obesity induces sperm infertility. During the process of sperm maturation in the epididymis, the sperm function can be modulated by changes in the expression levels of exosomes, which are mainly secreted by the EECs lining the lumen of caput epididymis. Obesity can alter the epididymal microenvironment by increasing the OS and ROS generation. Such changes upregulate the stress-induced ER response in the EECs. Furthermore, ER stress can alter the quantities and contents of the exosomes in the epididymal fluid which may affect the sperm function, finally leading to male subfertility or infertility

The exosomes are critical for the regulation of sperm maturation and the maintenance of male fertility. We show here that the protein expression profiles of the epididymis-derived exosomes were largely changed in obese mice and several ER proteins were identified to be distributed in the exosomes of obese mice, which indicated a relationship between the ER stress and exosome packaging in the epididymal epithelial cells (EECs). Moreover, our results also showed that oxidative stress-induced ER stress in EECs can upregulate the quantity of the secreted exosomes as well as modulate the protein composition of the exosomes, which may be relevant to sperm maturation and sperm fertility (Fig. 7). Therefore, our results initially demonstrated that ER stress in the epididymal epithelium was a key factor in regulating the biogenesis and packaging of the exosomes, which appeared to facilitate stress adaptation in the epididymal microenvironment during sperm maturation.

The status of oxidative stress is dependent on the extent of imbalance between the oxidant and antioxidant system. Oxidative stress is imposed by an attenuated antioxidant system and the overproduction of ROS [14, 16, 50]. Other changes accompanying the increases in the OS level include increases in the levels of MDA in the serum, body fluid and reproductive tissues, and declines in the reductive glutathione GSH levels due to its transformation into its oxidized glutathione GSSG form [51, 52]. Many studies on male infertility revealed that excessive ROS levels accounted for increases in OS status which can directly damage the sperm membrane, mitochondria and DNA, thereby resulting in impaired sperm motility and male infertility [15, 16, 18, 53]. On the other hand, several studies already showed that obesity can induce the systemic and local OS both in humans and murine animals. This condition alters oxidative phosphorylation, induces chronic inflammation, hyperleptinemia, endothelial and mitochondrial dysfunction, etc. [18, 54–56]. However, the mechanism is still unclear of OS-induced dysfunction in sperm maturation during its movement through the epididymis. To address this question, we established an obese mouse model and investigated how OS impaired the sperm fertility. As expected, the obese mice had increased MDA expression levels in the epididymal fluids whereas the GSH/GSSG ratio declined in the caput epididymis, coupled with impaired sperm motility, indicating that obesity can induce rises in the OS level in the epididymal microenvironment, subsequently leading to the impairment of the sperm quality.

Epididymis-derived exosomes (epididymosomes) secreted from EECs into the epididymal lumen, play several important roles in the maintenance of the epididymal microenvironment. These exosomes can communicate with the spermatozoa through sharing with their protein and non-coding RNA content. As reported, both of these constituents can be altered by changes in the OS levels. Such changes stem from variations including heat stress, metabolic stress, hypoxia, oxidative stress, UV radiation, etc. [50, 57, 58]. Besides, in vitro studies also showed that the protein and RNAs cargos of exosomes or extracellular vesicles were changed under the inflammatory or oxidative stress condition [50, 59–61]. More importantly, it was reported that both the quantity and inclusion of epididymosomes were changed dynamically in response to the paternal exposure to different stressful conditions [2]. Thus, we speculated that the characteristics of the exosomes may be altered while the epididymal microenvironment composition is altered by changes in the OS status. Such alterations can lead to disruption of normal exchanges between the EEC and sperm through changes in the exosome content that can interfere with the sperm maturation process.

To probe for the obesity-induced changes in the proteomic composition of the exosomes in the epididymal microenvironment, we separated and purified the exosomes from the mouse epididymal fluid. Currently, there are five typical methods used for the extraction of exosomes, including ultracentrifugation-based isolation techniques, size-based isolation techniques, immunoaffinity capture-based techniques, exosome precipitation and microfluidics-based isolation techniques [6, 7, 62]. Because the method of polymer-based precipitation was simple to perform and suitable for enrichment of exosomes from a small sized sample [6], we employed this method for exosome separation. When obtained, the isolated exosomes were further identified under an electron microscope in conjunction with nanoparticle tracking and Western blot analyses. This procedure was employed to confirm their integrity, size, and purity. The results showed that our preparation was adequate for performing relevant proteomic analysis.

In the proteomic analysis, we totally identified 4,338 proteins. The protein expression profiles showed that the marker proteins of exosome, such as CD9, flotillin 1, TSG101 and CD63, were adequately enriched in the isolated epididymis-derived exosomes both in the control mice and obese mice, whereas the exosome negative protein such as calnexin was not identified in both groups [63, 64]. The proteins screening by proteomics, combined with the analyses of electron microscope, nanoparticle tracking and Western blot, further demonstrated that the isolated exosomes had high purity and excellent quality. Among the 4338 proteins, there were 202 DEPs in the exosomes between the control mice and obese mice. Interestingly, we found an enrichment of immune response related proteins with the maximum difference among the upregulated proteins. Obesity is widely perceived as contributing to the chronic inflammatory state in association with the increased pro-inflammatory cytokines in local reproductive system, which generally results in the disruption of spermatogenesis and sperm maturation by the overproduction of ROS in the seminiferous epithelium and epididymal epithelium [18]. Meanwhile, among the DEPs, there were lots of proteins involved in the metabolism, corresponding to obesity related metabolic disorder. Accordingly, such changes in the protein composition in exosomes coincided with the known alterations that account for both the etiology and clinical manifestations in obesity.

It is worth noting that the epididymosome can transport their cargos to the spermatozoa located in the post-acrosomal domain of the sperm head [9, 10], and then alter regulation of sperm fertility [65]. Herein, the proteins related to the regulation of reproduction, such as acrosomal protein SP-10, sperm acrosome-associated protein 7 (SPACA7) and zona pellucida sperm-binding protein 3 receptor, were also screened in the current proteomic study.

Furthermore, according to the GO analysis of the DEPs, these proteins were mostly enriched in the function termed “respond to stress”. It is well understood that the unfold protein reaction (UPR) in the ER was specifically activated when the ER stress was induced by the multiple stresses, including high temperature, infection, toxins, diseases and ROS, to overcome the accumulation of mis-folded and unfolded proteins in ER [29, 35, 45, 46, 66]. In our proteomic data, we found that the proteins localized in the ER and associated with stress were significantly changed in the exososmes from obese mice, e.g., HSP90 was involved in the activation of the cytosolic heat-shock response (HSR) [45] and the RRBP1, CRELD2 and PDIA3 were related to the ER stress. Although the ER is the primary compartment for protein synthesis, folding and secretory activity [45, 67], there are very limited reports showing that ER stress can change the quantity and content of the exosomes. Therefore, our aim was to elucidate how ER stress in the EECs affects the exosome biogenesis and cargo packaging, especially the expression level changes in the ER proteins of RRBP1, CRELD2 and PDIA3.

RRBP1, CRELD2 and PDIA3 are mainly localized in the ER and involved in the regulation of ER function [30, 34, 39]. For instance, RRBP1 is defined as a ribosome-binding protein and critical for the transport and secretion of the proteins [30–32]. Recent studies showed that RRBP1 is associated with the regulation of the UPR signaling molecules, such as the formation of the initial complex Bip/Grp78 in the initiation of ER stress [29]. CRELD2 is a member of the CRELD (cysteine rich with epidermal growth factor-like domains) family and was firstly identified as a novel mediator of ER stress due to its promoter containing an ATF6 response element [34]. CRELD2 was reported to be involved in a kidney disease induced by ER stress and tumor development mediated by the ROCK-PERK-ATF4 signaling pathway [36, 68]. PDIA3 is a member of the PDI (protein disulfide isomerase) family that catalyzes the redox of disulfide bonds in substrates to maintain protein folding and activity [39, 40]. The PDI family can mediate the activities of the Bip compound, ATF6, PERK and IRE1α by redox regulation [66], which was tightly associated with the ER stress. Thus, in the current study, the differential expression of RRBP1, CRELD2 and PDIA3 in exosomes may indeed attribute to obesity-induced ER stress.

In eukaryotic cells, exosomes are initially formed as intraluminal vesicles through multivesicular compartments of the endocytic pathway and they exhibit different populations owing to their origin from different subcellular sites and different containing cargoes [5, 69]. Recent research on intracellular trafficking of exosomes found that the exosomes with markers CD9 and CD63 are all originated from the ER and trafficked to their residency compartment [69]. Our proteomic and Western blot data showed that the protein expression levels of both CD9 and CD63 in exosomes significantly increased in obese mice. Such changes undoubtedly indicated the increased biogenesis and secretion of exosomes, especially exosome formation originated from the ER. Therefore, our findings provide meaningful evidences that the ER stress can promote exosome formation originated from ER and change the proteins cargoes in the exosome subpopulations. In addition, it also documents a potential mechanism that describes how OS-induced environmental challenges impair sperm fertility through accentuating ER stress in the epididymis and compromising exosome function during sperm maturation.

## Conclusion

In the current study, we employed comparative proteomics analysis to investigate the differential expression patterns of proteins in exosomes derived from the epididymis of obese mice. The results showed that elevated ER stress accounted for the differential ER protein expression pattern in exosomes. Such changes may play key roles in regulating sperm maturation. Moreover, our results also initially demonstrated that the biogenesis and cargo packaging of the exosomes were closely associated with OS-induced ER stress in EECs. To further probe for the biological effects of these proteins whose expression levels were impacted by exposure to elevated OS levels in epididymal microenvironment, may unravel how elevated ER stress in EECs can affect sperm quality and male fertility via exosomes. 

## Supplementary Information

**Additional file 1.** 

**Additional file 2.** 

**Additional file 3.** 

**Additional file 4.** 

**Additional file 5.** 

**Additional file 6.** 

**Additional file 7.** 

**Additional file 8.** 

**Additional file 9.** 

**Additional file 10.** 

**Additional file 11.** 

**Additional file 12.** 

**Additional file 13.** 

**Additional file 14.** 

**Additional file 15.** 

## Data Availability

The datasets used and analyzed during the current study are available from the corresponding author on reasonable request.

## Abbreviations

EEC: epididymal epithelial cell
OS: oxidative stress
LC-MS: Liquid chromatography tandem mass spectrometry
GO: Gene Ontology
DEPs: differentially expressed proteins
ER: endoplasmic reticulum
PA: palmitic acid
CASA: computer-assisted sperm analysis
SSA: sulfosalicylic acid
PBS: Phosphate Buffer Saline
TEM: transmission electron microscopy
NTA: Nanoparticle tracking analysis
PSMs: Peptide Spectrum Matches
FDR: false discovery rate
PFA: paraformaldehyde
IHC: Immunohistochemistry
MDA: malondialdehyde
UPR: unfolding protein response
IRE1α: inositol-requiring enzyme 1α
PERK: protein kinase R-like ER kinases
HSR: heat-shock response
RRBP1: ribosome-binding protein 1
CRELD: cysteine rich with epidermal growth factor-like domains
PDI: protein disulfide isomerase.
