# Supplementary material for: Endoplasmic reticulum stress increases exosome biogenesis and packaging relevant to sperm maturation in response to oxidative stress in obese mice
Source: Reprod Biol Endocrinol. 2022 Nov 21;20:161. doi: 10.1186/s12958-022-01031-z (PMC9677646; doi:10.1186/s12958-022-01031-z)

Figure-4A PDIA3/RRBP1/CRELD2/TSG101

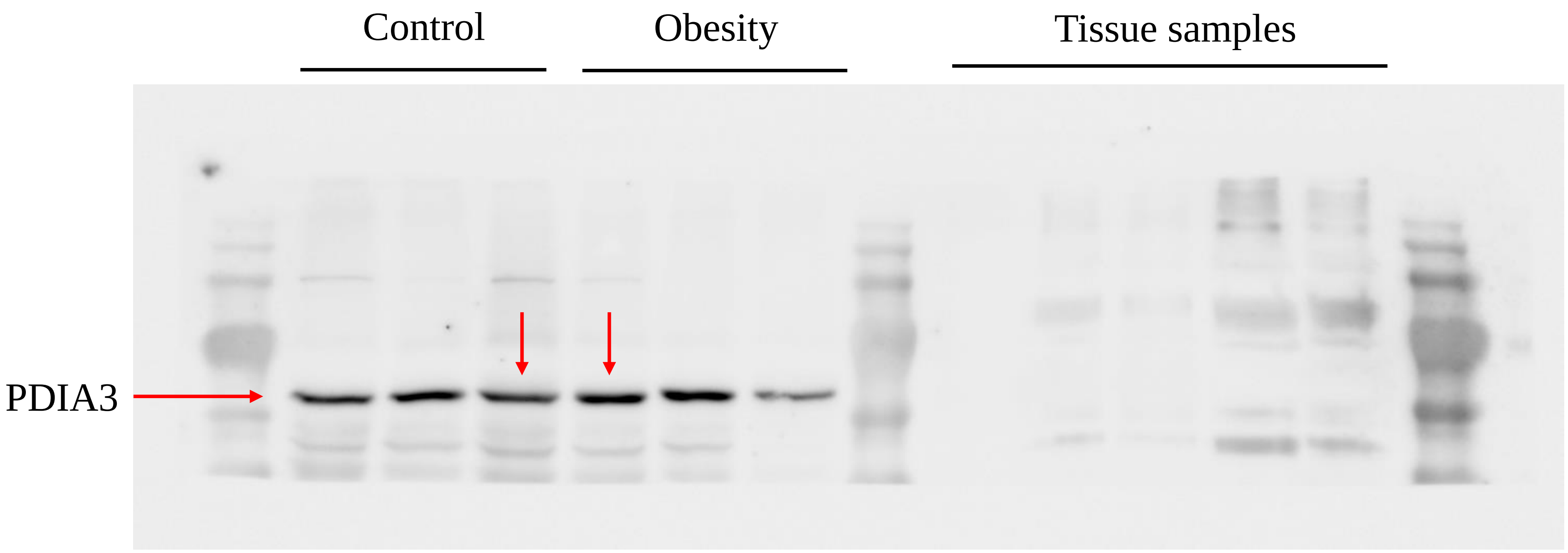

Figure-4A PDIA3/RRBP1/CRELD2/TSG101

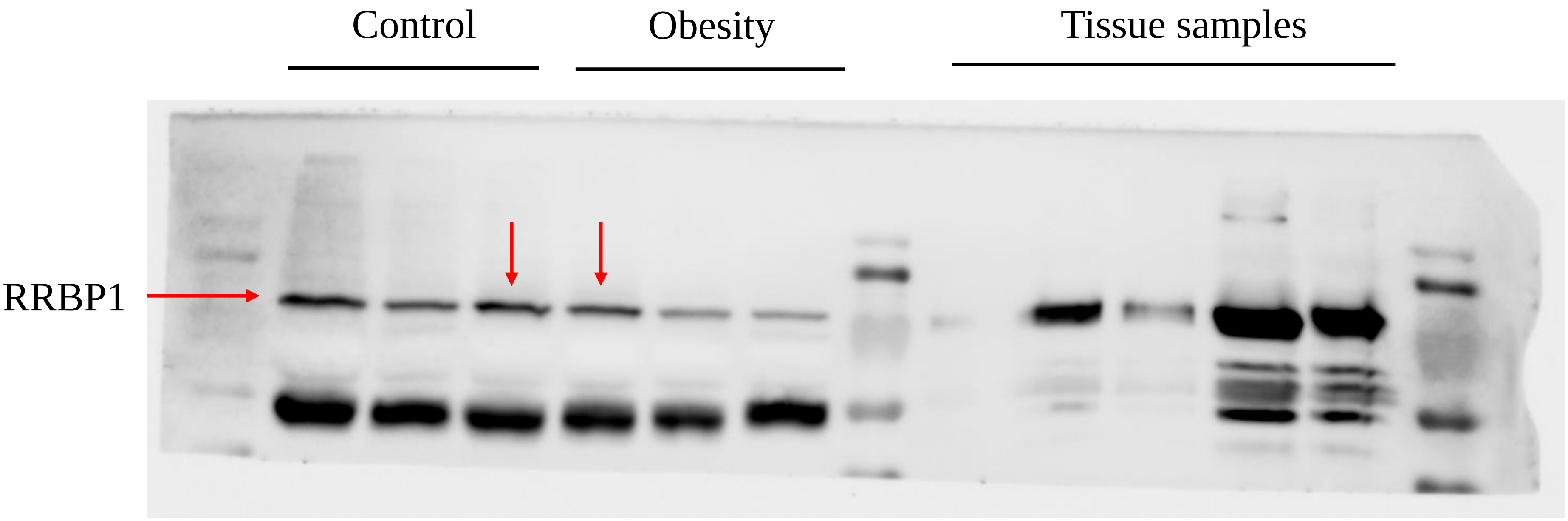

Figure-4A PDIA3/RRBP1/CRELD2/TSG101

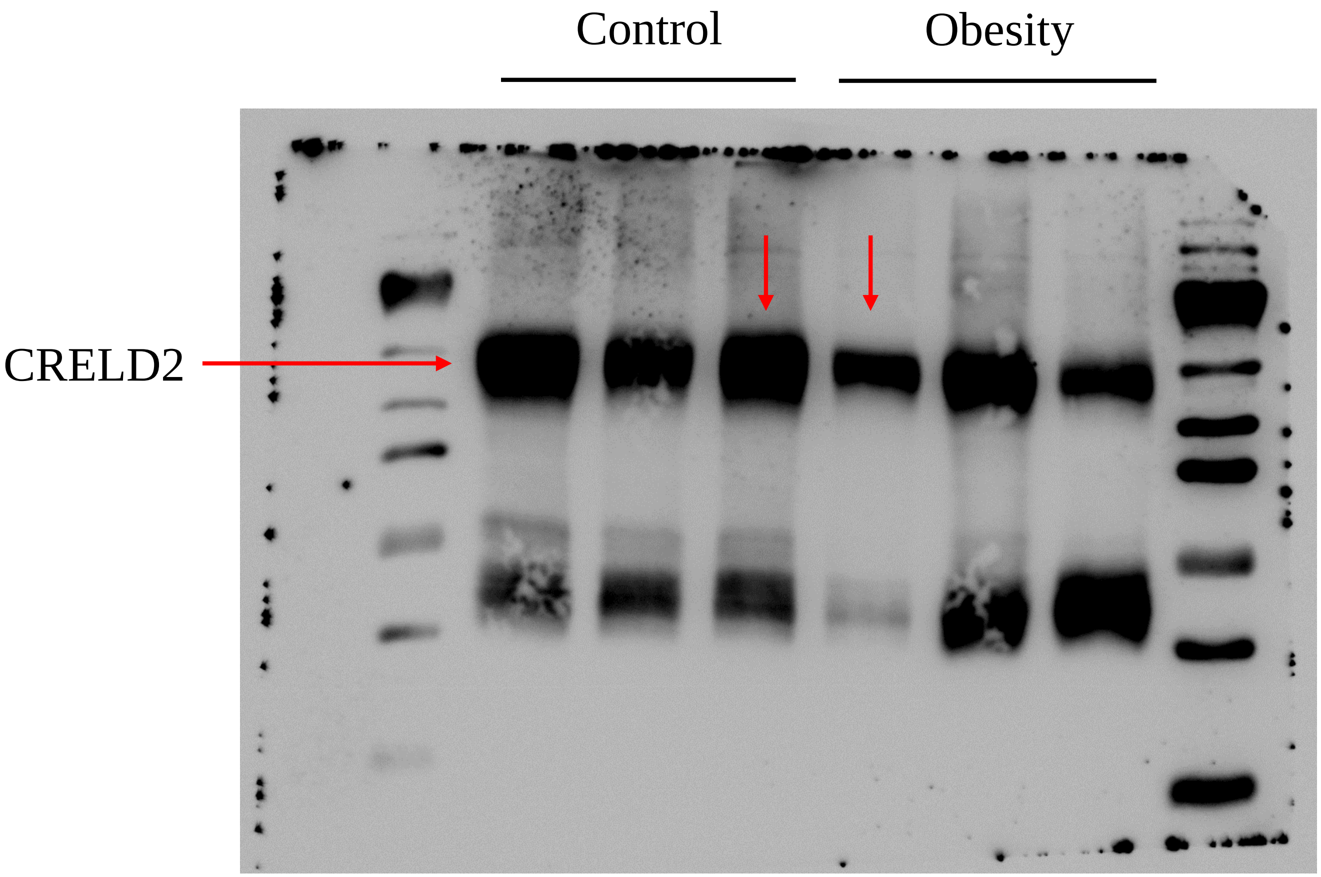

Figure-4A PDIA3/RRBP1/CRELD2/TSG101

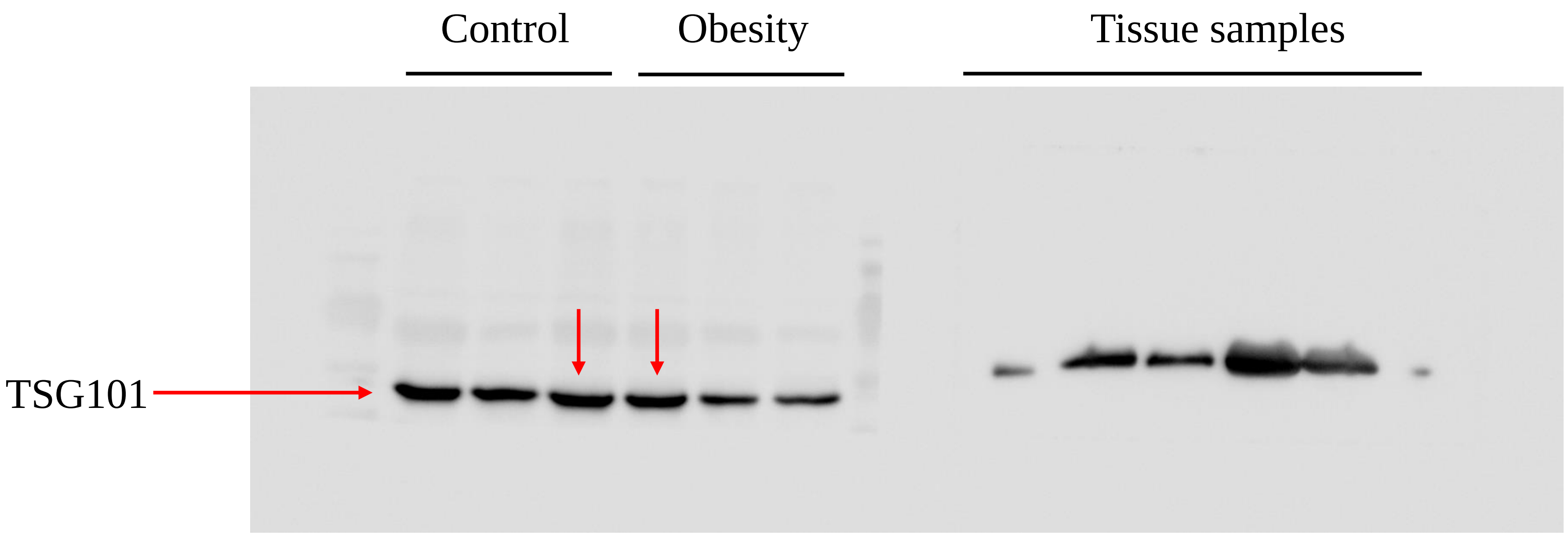

Supplement: Supplementary file 2 — Additional file 2. [file 12958_2022_1031_MOESM2_ESM.pdf]
