# Supplementary material for: Endoplasmic reticulum stress increases exosome biogenesis and packaging relevant to sperm maturation in response to oxidative stress in obese mice
Source: Reprod Biol Endocrinol. 2022 Nov 21;20:161. doi: 10.1186/s12958-022-01031-z (PMC9677646; doi:10.1186/s12958-022-01031-z)

Figure-5A:PDIA3/β-actin

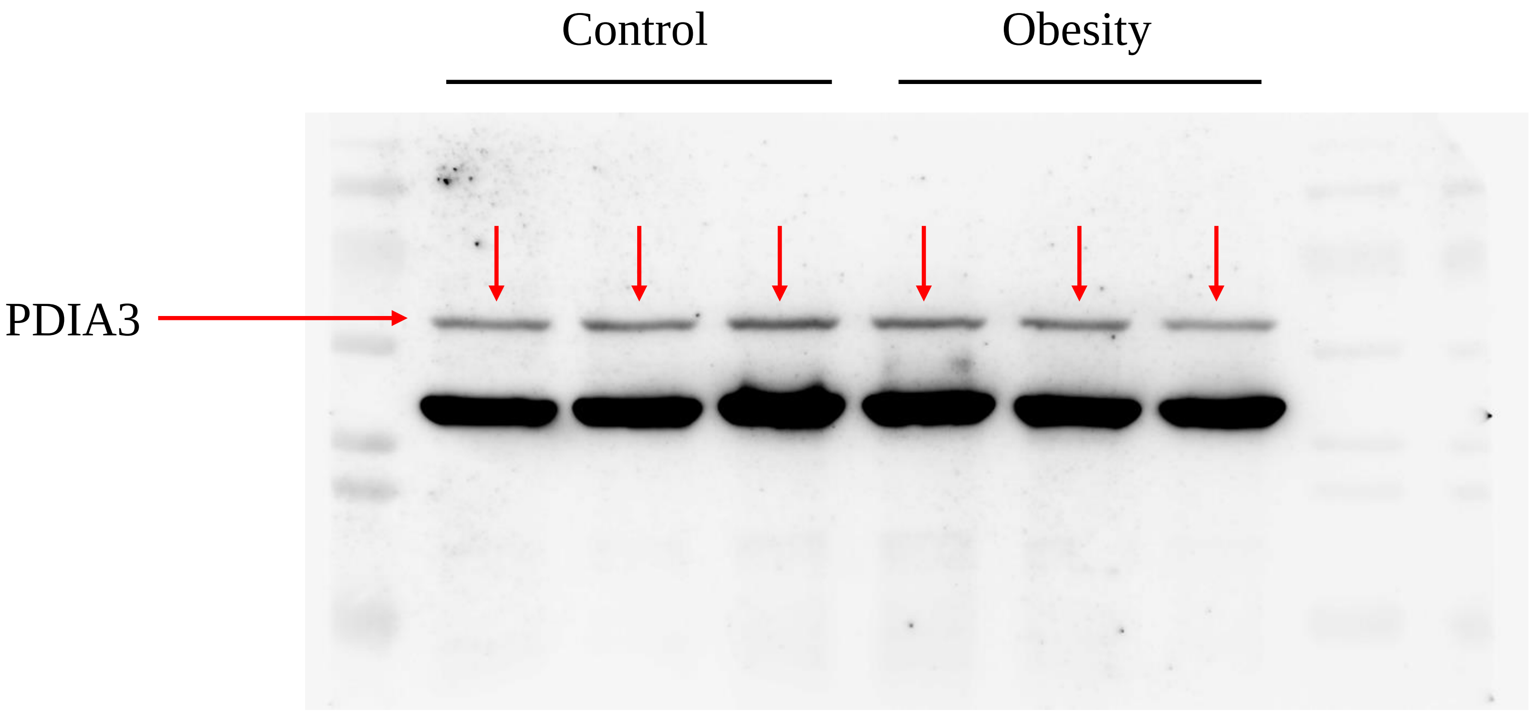

Figure-5A:PDIA3/β-actin

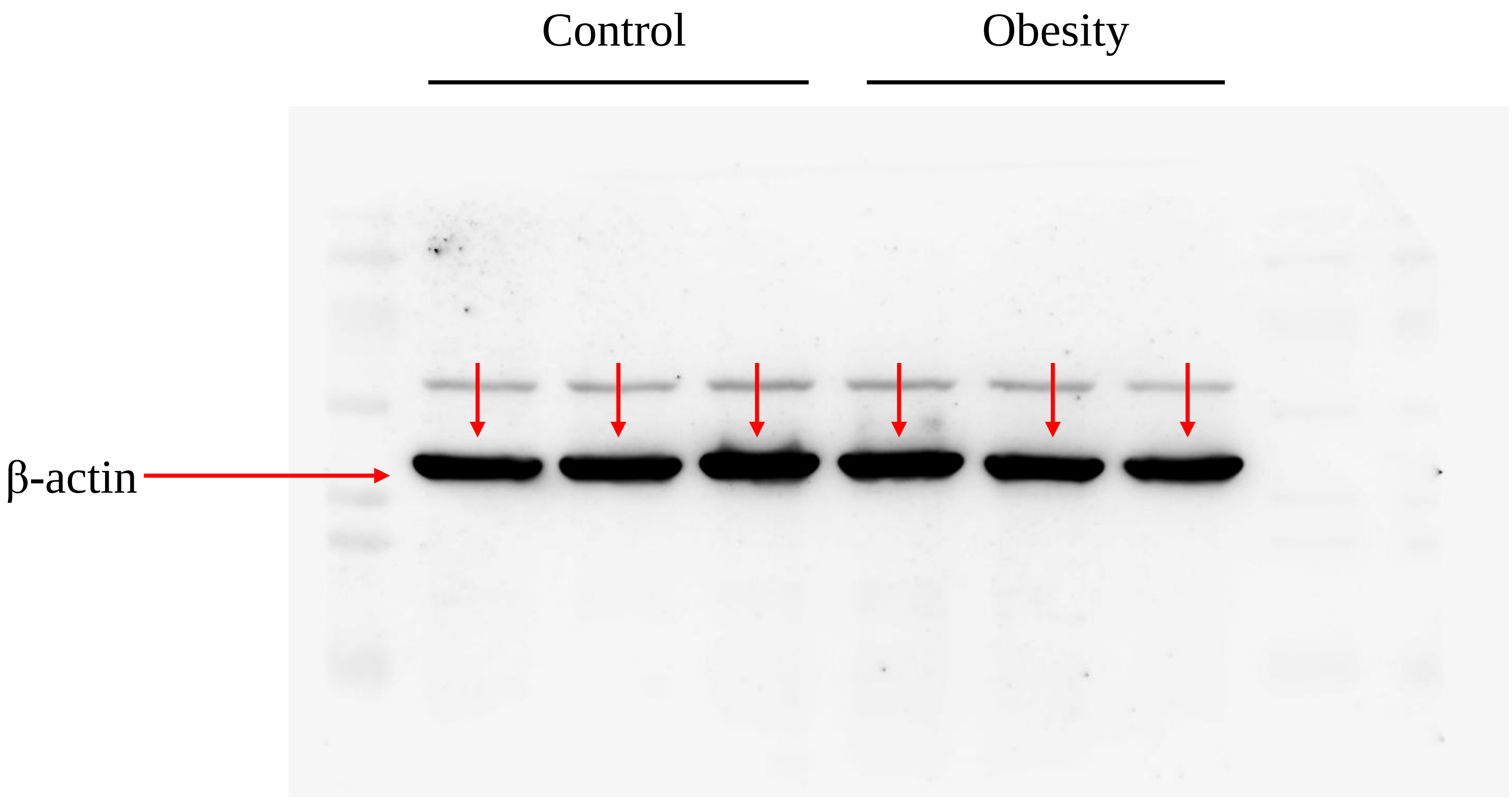

Figure-5A:RRBP1/β-actin

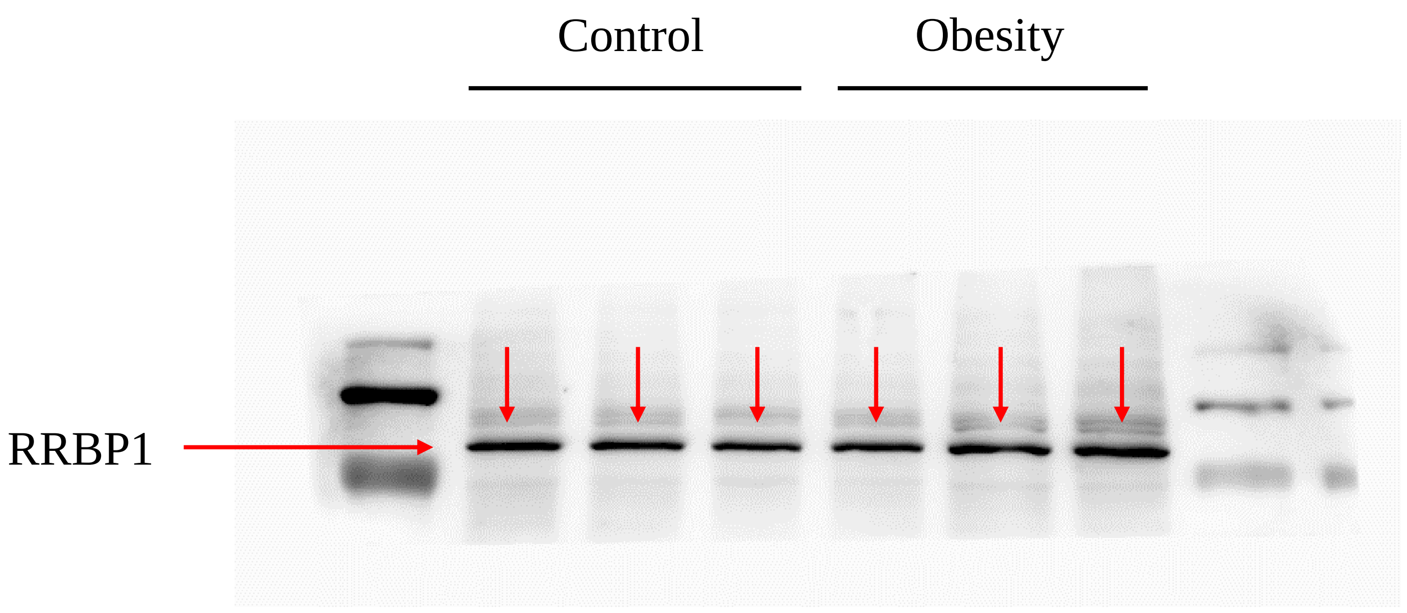

Figure-5A:RRBP1/β-actin

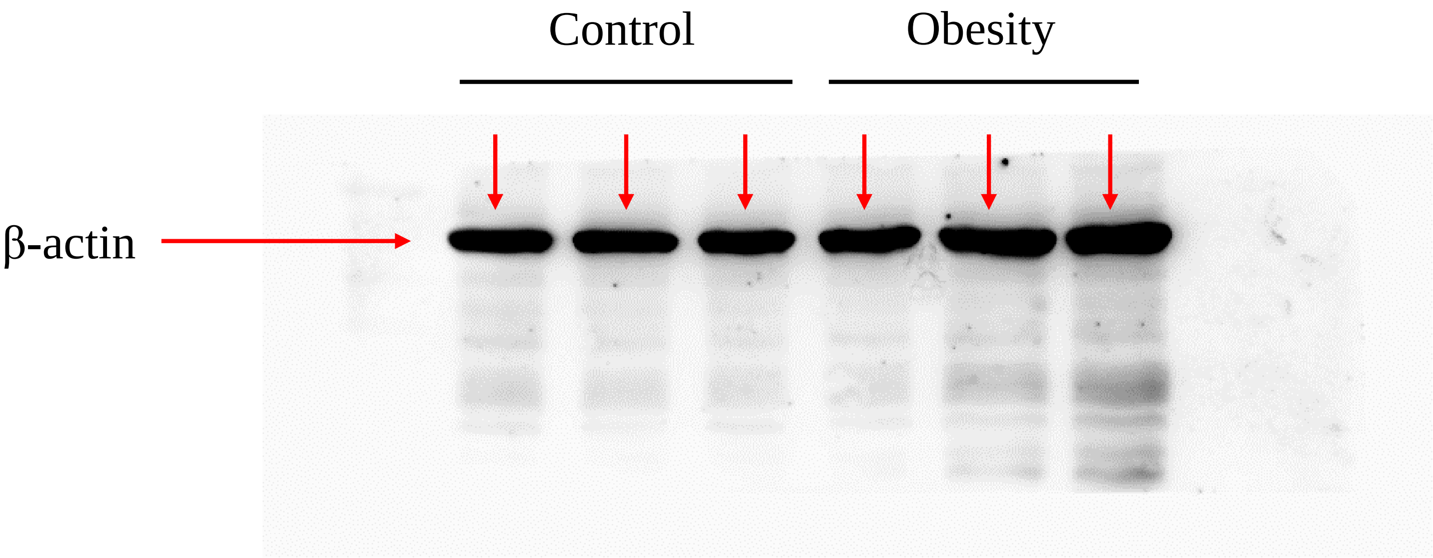

Supplement: Supplementary file 3 — Additional file 3. [file 12958_2022_1031_MOESM3_ESM.pdf]
