# Supplementary figures and images for: Endoplasmic reticulum stress increases exosome biogenesis and packaging relevant to sperm maturation in response to oxidative stress in obese mice
Source: Reprod Biol Endocrinol. 2022 Nov 21;20:161. doi: 10.1186/s12958-022-01031-z (PMC9677646; doi:10.1186/s12958-022-01031-z)

Figure-5C:CRELD2/CD63/ $\beta$ -actin

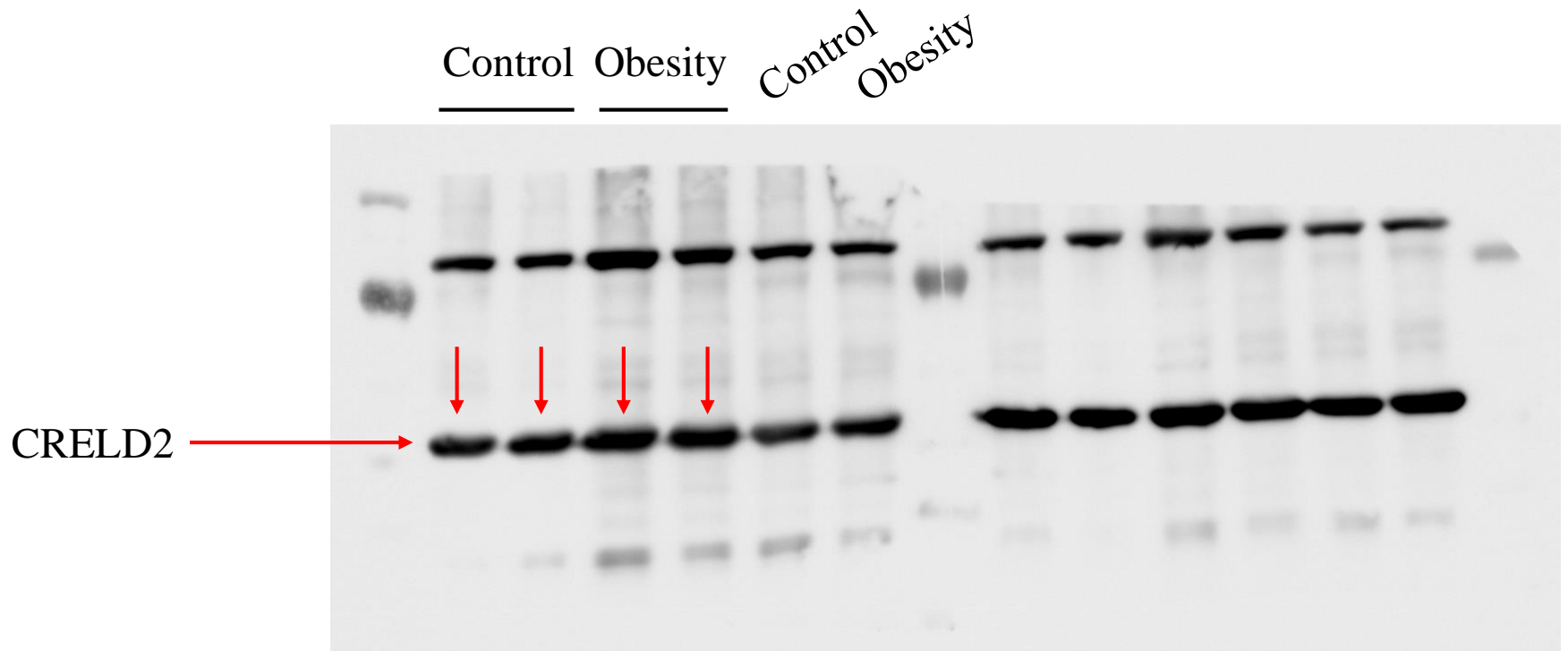

Figure-5C:CRELD2/CD63/ $\beta$ -actin

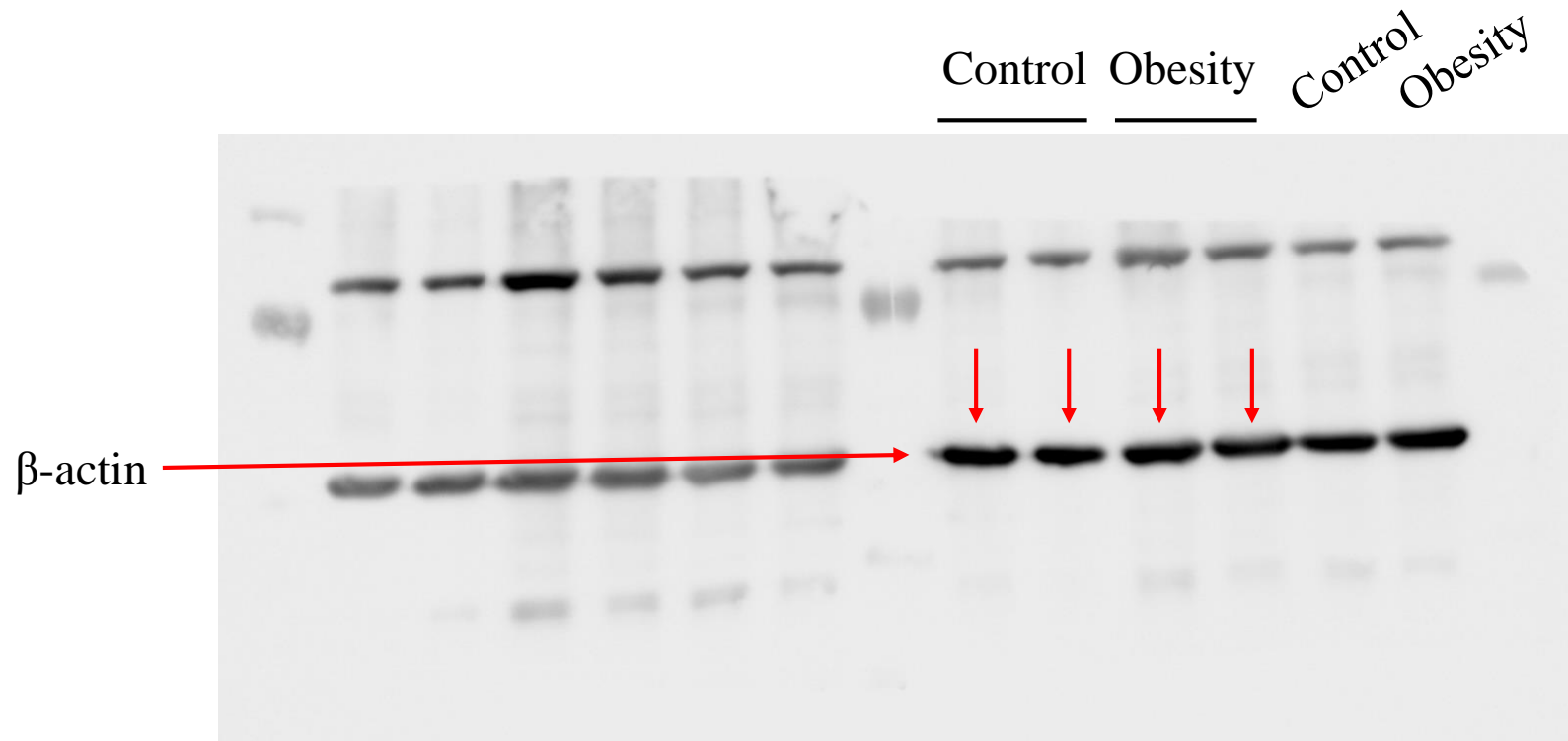

Figure-5C:CRELD2/CD63/ $\beta$ -actin

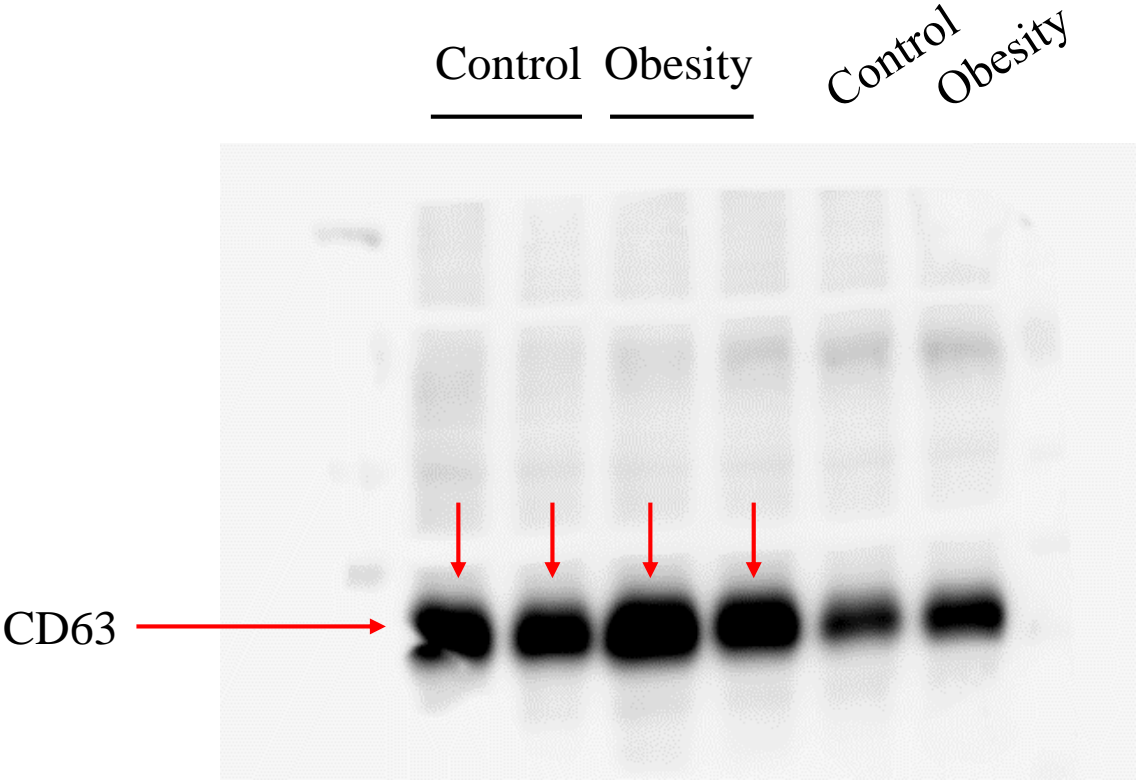

Supplement: Supplementary file 4 — Additional file 4. [file 12958_2022_1031_MOESM4_ESM.pdf]

Figure-5G:CD63/TSG101

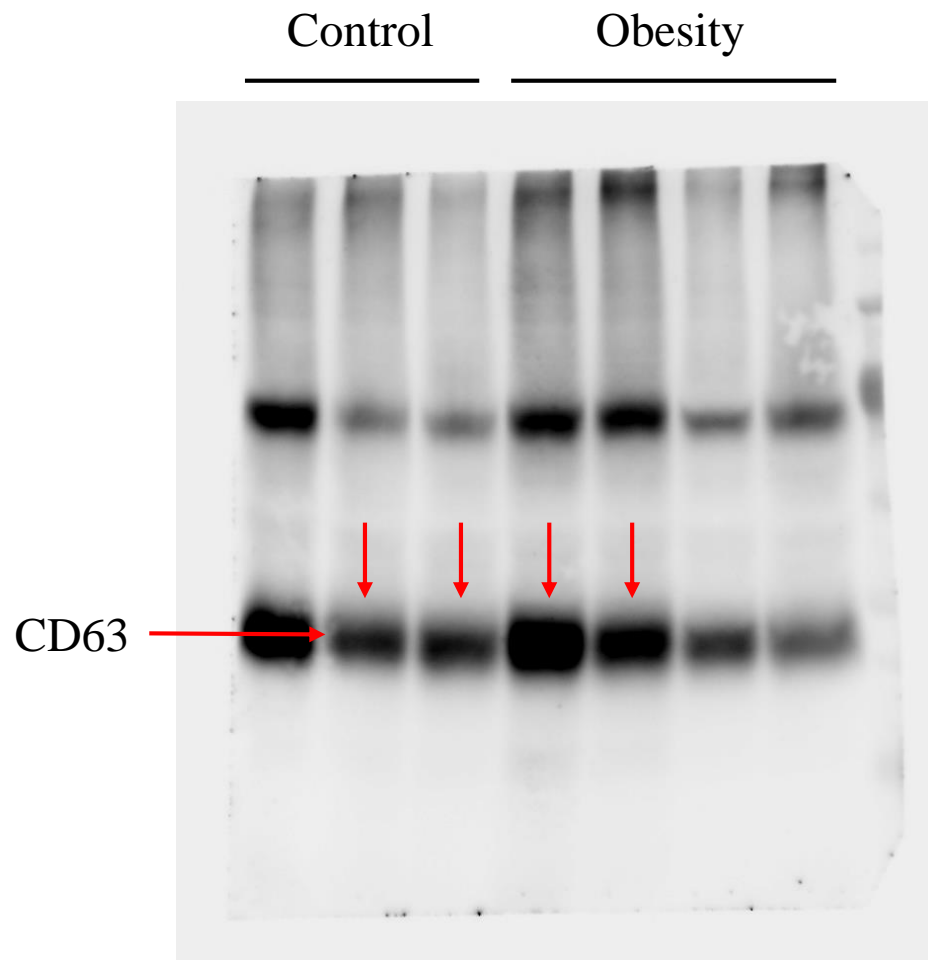

Figure-5G:CD63/TSG101

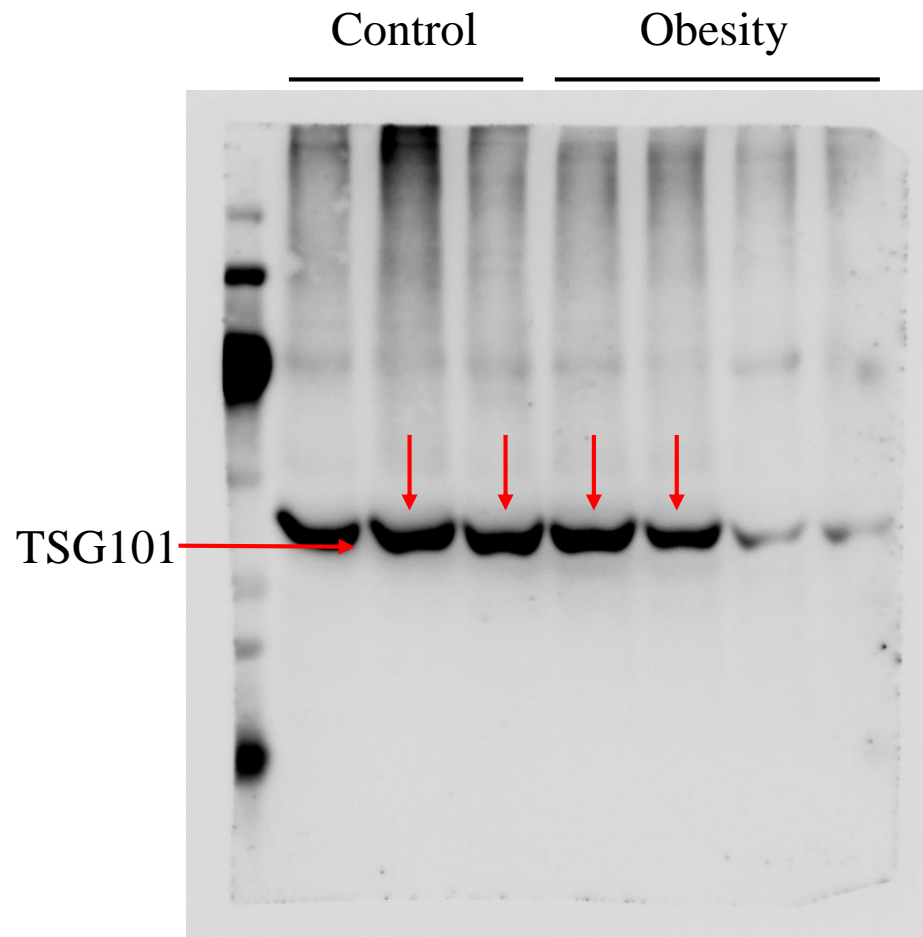

Supplement: Supplementary file 6 — Additional file 6. [file 12958_2022_1031_MOESM6_ESM.pdf]

Figure-6G:CRELD2/CD63/ $\beta$ -actin

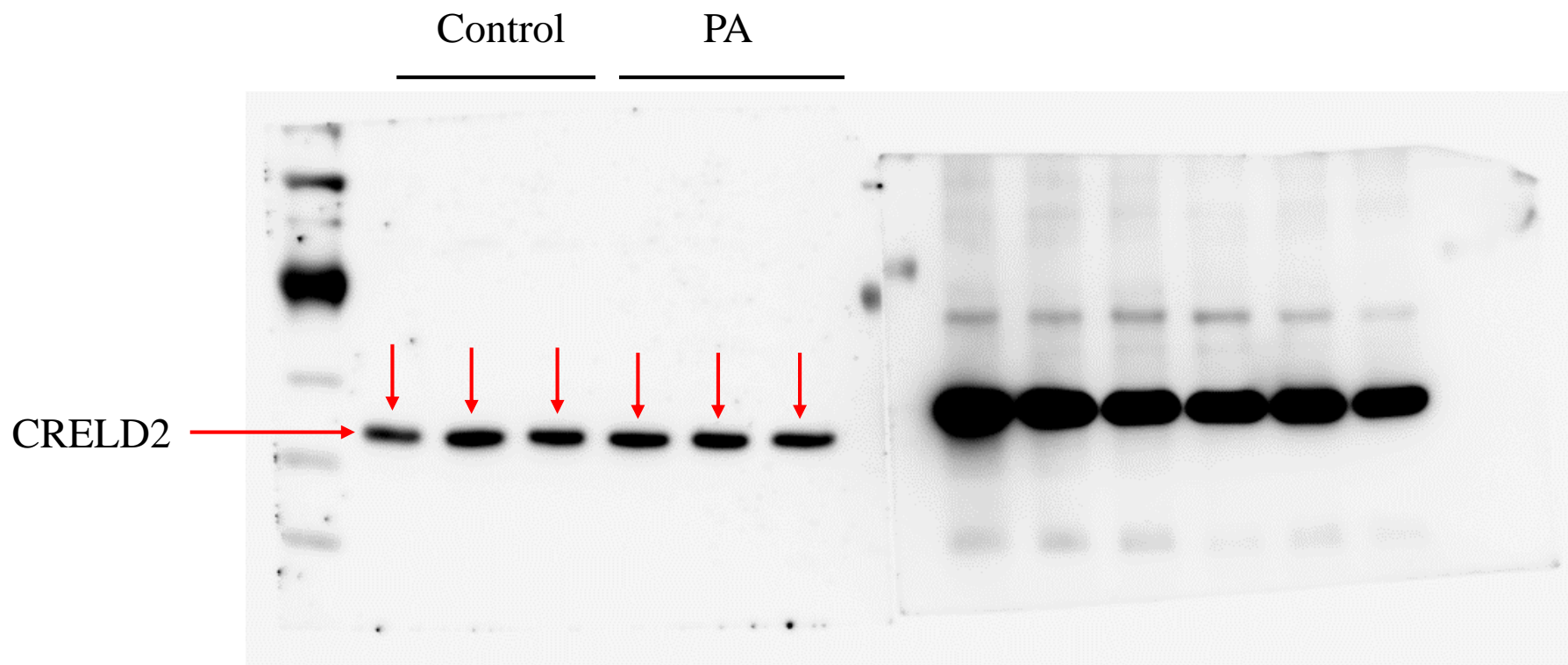

Figure-6G:CRELD2/CD63/ $\beta$ -actin

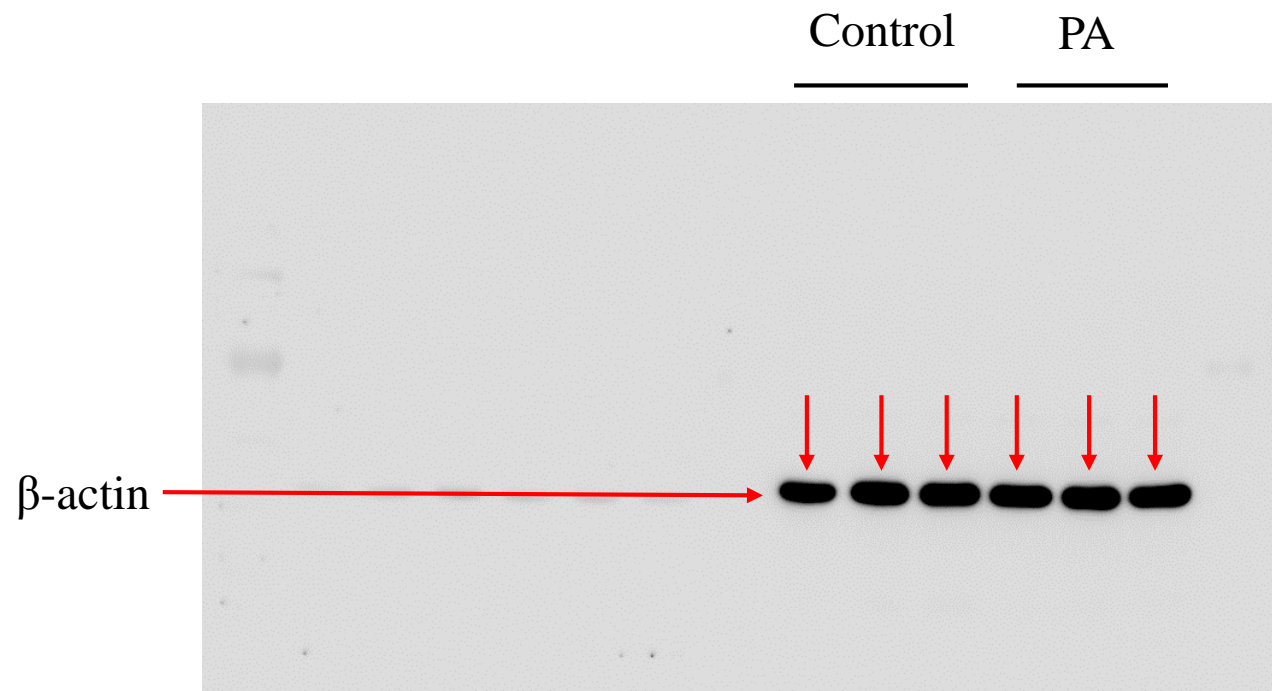

Figure-6G:CRELD2/CD63/ $\beta$ -actin

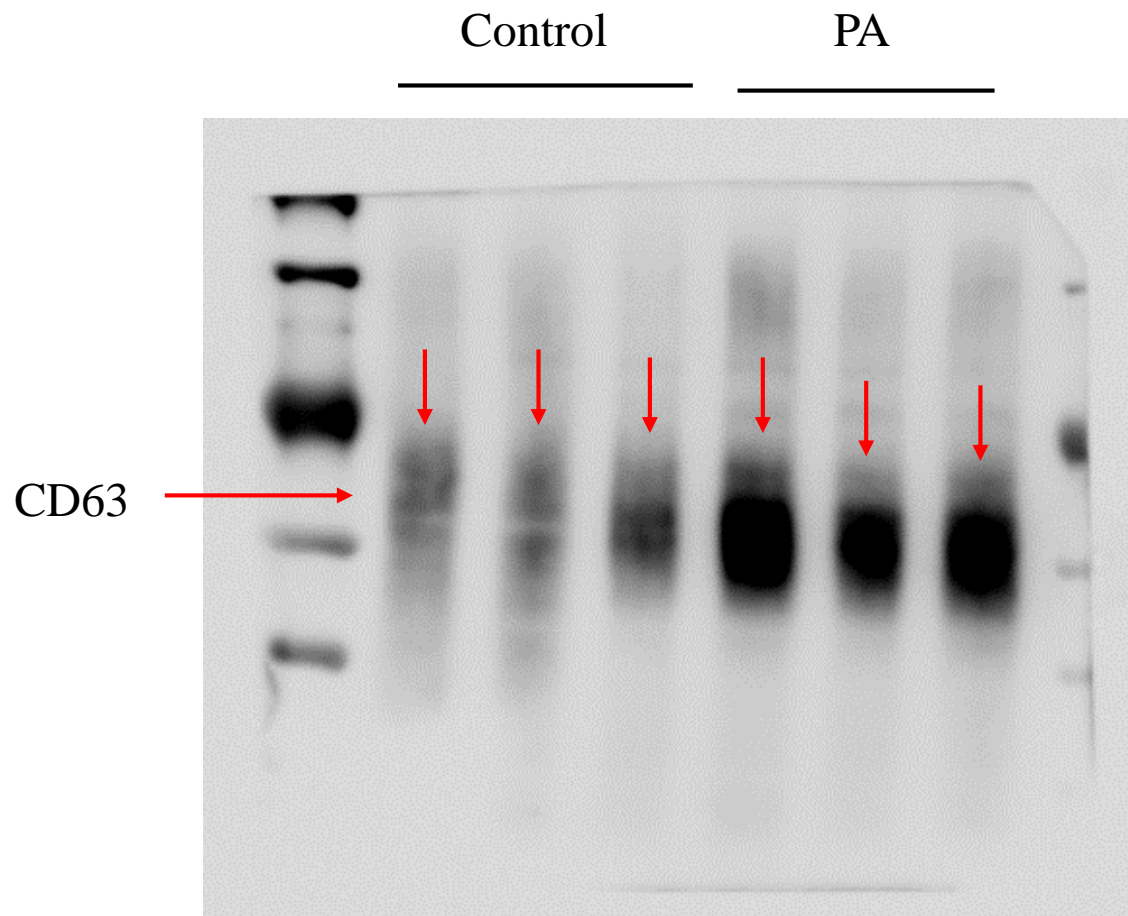

Supplement: Supplementary file 15 — Additional file 15. [file 12958_2022_1031_MOESM15_ESM.pdf]
