# Supplementary material for: Endoplasmic reticulum stress increases exosome biogenesis and packaging relevant to sperm maturation in response to oxidative stress in obese mice
Source: Reprod Biol Endocrinol. 2022 Nov 21;20:161. doi: 10.1186/s12958-022-01031-z (PMC9677646; doi:10.1186/s12958-022-01031-z)

Figure-6E:PDIA3/RRBP1/β-actin

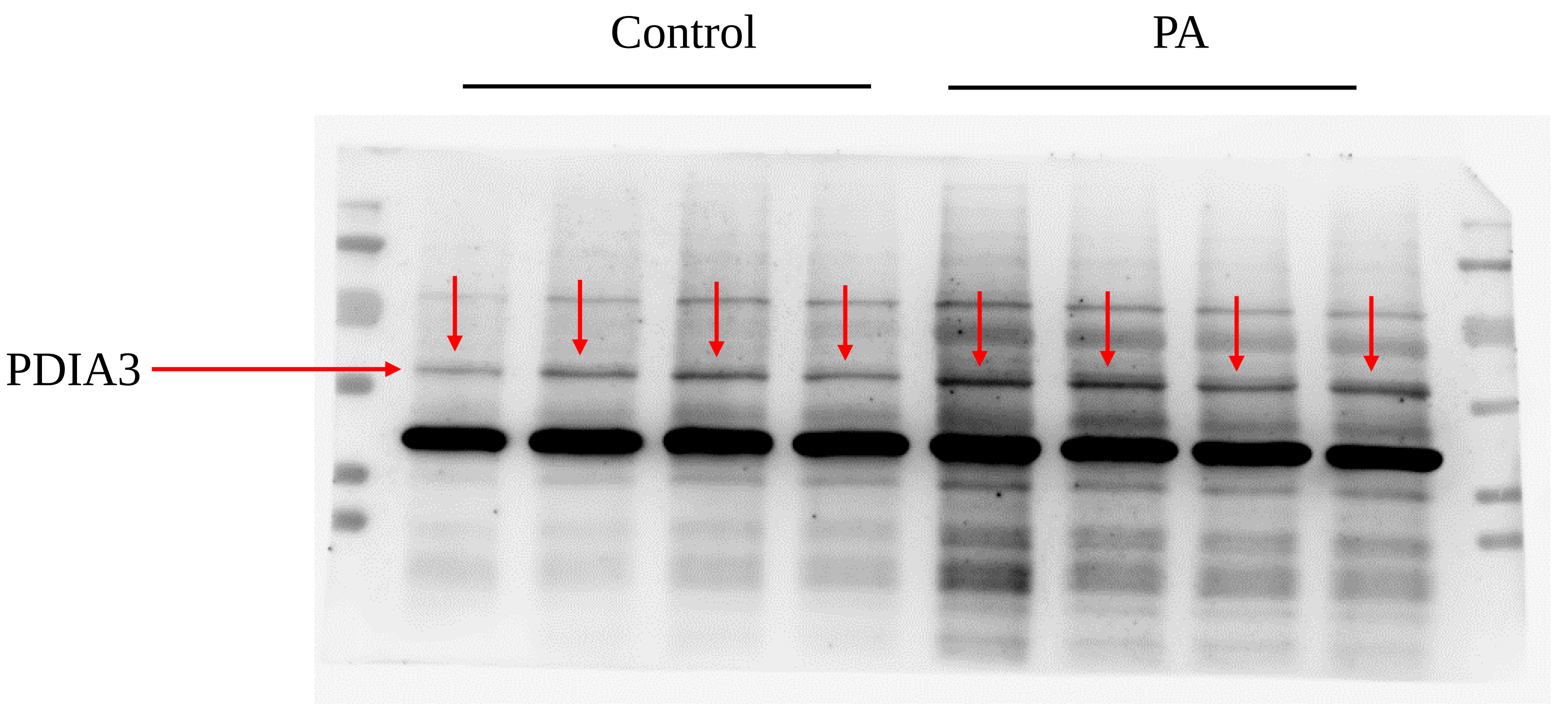

Figure-6E:PDIA3/RRBP1/β-actin

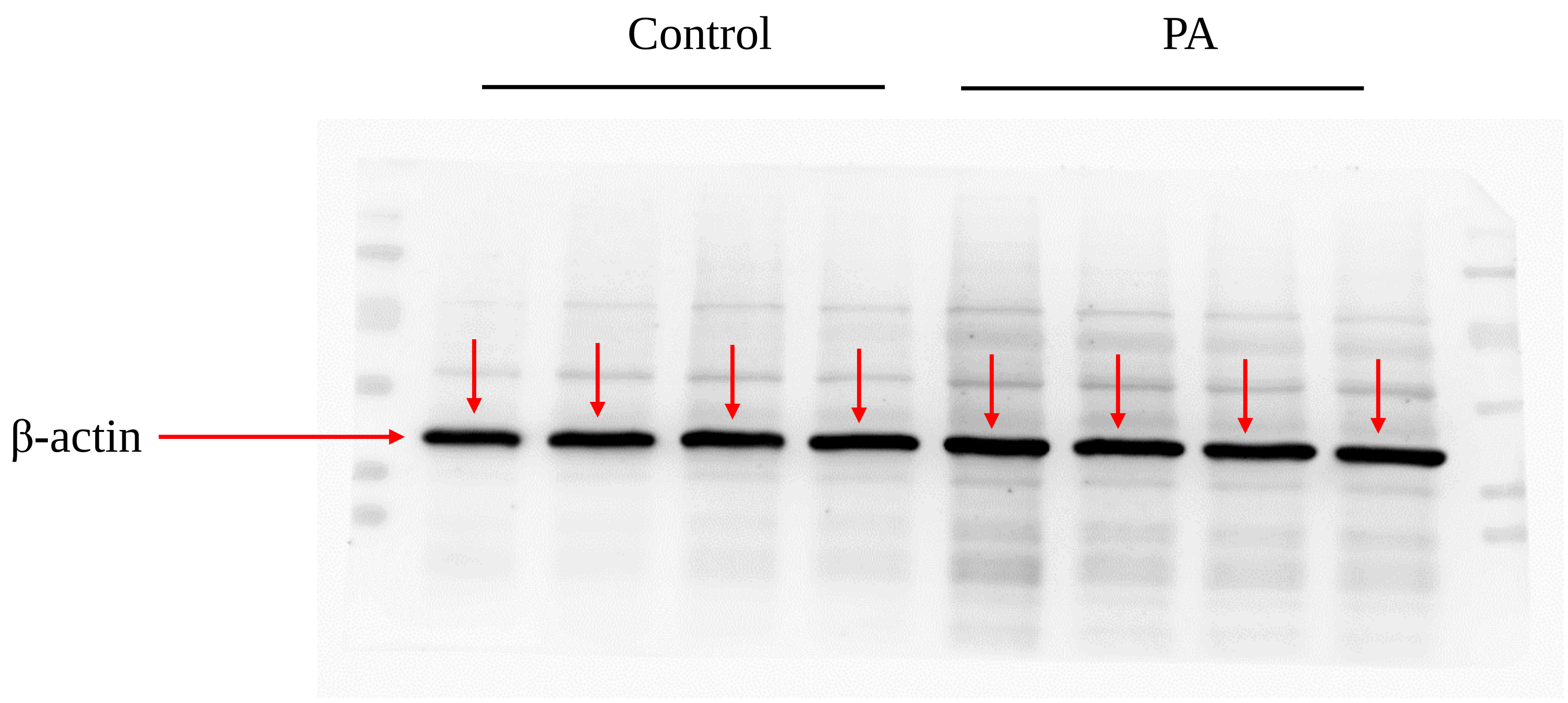

Figure-6E:PDIA3/RRBP1/β-actin

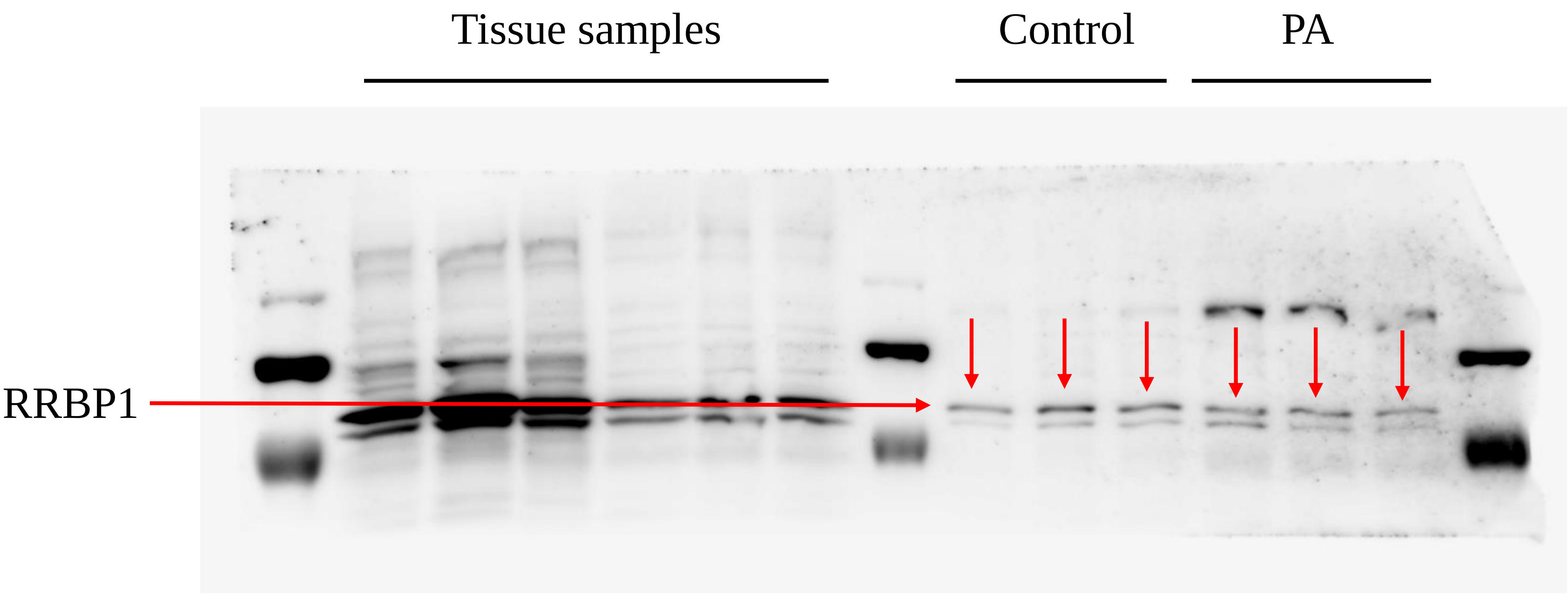

Figure-6E:PDIA3/RRBP1/β-actin

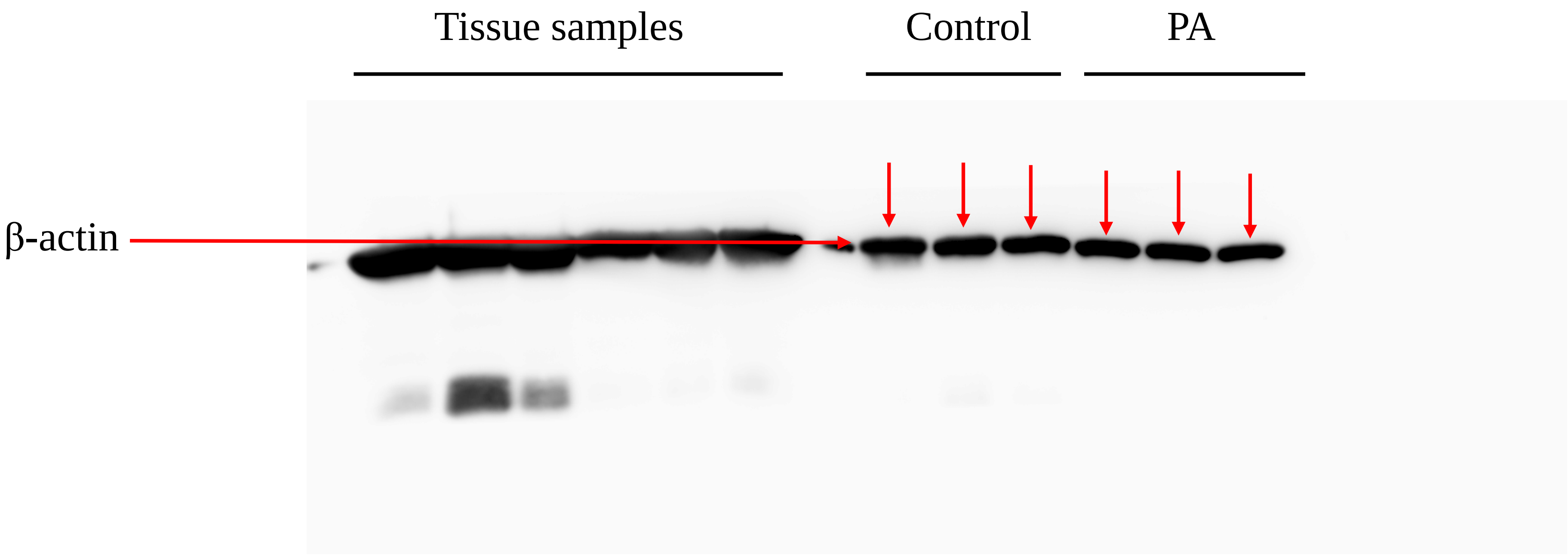

Supplement: Supplementary file 14 — Additional file 14. [file 12958_2022_1031_MOESM14_ESM.pdf]
